# Exosome-equipped TNF antisense oligodeoxynucleotide or 2-deoxy-D-glucose ameliorated nonalcoholic steatohepatitis by modulating superoxide dismutase 1 in mice

**DOI:** 10.1016/j.redox.2025.103488

**Published:** 2025-01-03

**Authors:** Fei He, Wei Du, Yingying Liu, Yuwei Ling, Ming Xu, Jingjing Liu, Ping Song, Zhiqiang Fang, Zhensheng Yue, Juanli Duan, Lin Wang

**Affiliations:** Department of Hepatobiliary Surgery, Xijing Hospital, Fourth Military Medical University, 710032, Xi'an, China

**Keywords:** Nonalcoholic steatohepatitis, Exosomes, Macrophage, TNF antisense oligodeoxynucleotide, 2-Deoxy-d-glucose, Sod1

## Abstract

Inflammatory mediators tumor necrosis factor (TNF) and interleukin 1 beta (IL1β), primarily derived from hepatic macrophages in the liver, play a crucial role in the progression of nonalcoholic steatohepatitis (NASH). Meanwhile, intravenously injected exosomes are mainly distributed in the liver and predominantly taken up by hepatic macrophage. Herein, we aimed to evaluate the feasibility of targeted inhibition of TNF and IL1β expression in hepatic macrophages via exosomes as a potential therapeutic strategy for NASH. In this study, we demonstrated that antisense oligodeoxynucleotide targeting TNF (ASO-TNF) or 2-deoxy-d-glucose (2DG) effectively suppressed the expression of TNF and/or IL1β in macrophages. Exosomes loaded with ASO-TNF or 2DG were able to suppress the expression of TNF and/or IL1β in macrophages *in vitro* or *in vivo*. Furthermore, infusion of Exo/ASO-TNF or Exo/2DG significantly attenuated experimental steatohepatitis in choline deficient amino acid-defined (CDAA) or methionine and choline deficient (MCD) diet-fed mice. RNA-seq results showed that treatment with Exo/ASO-TNF or Exo/2DG significantly inhibited pro-inflammatory signaling pathways. Mechanistically, we observed that administration of Exo/ASO-TNF or Exo/2DG could attenuate NASH progression by up-regulating the expression of superoxide dismutase 1 (Sod1). Combined, our findings demonstrated that infusion of exosomes loaded with ASO-TNF or 2DG alleviated experimental steatohepatitis in murine models. Thus, infusion of exosomes loaded with anti-inflammatory agents holds promise as a potential therapeutic strategy for NASH treatment.

## 1 Introduction

Nonalcoholic fatty liver disease (NAFLD) has emerged as the most prevalent form of chronic liver disease, affecting over a quarter of the global population [[1], [2], [3]]. NAFLD varies from nonalcoholic fatty liver (NAFL) characterized by hepatocyte triglyceride accumulation, to the more severe nonalcoholic steatohepatitis (NASH). NASH is distinguished by hepatic steatosis, hepatic inflammation and fibrosis, with potential progression to cirrhosis and hepatocellular carcinoma [[1], [2], [3]]. However, there are still limited appropriate drugs specifically to treat NAFLD.

Hepatic macrophages are the largest population of innate immune cells in the liver [4]. It is well established that hepatic macrophage-derived inflammatory mediators play a crucial role in the progression of NAFLD. It has been observed that interleukin 1 beta (IL1β) derived from hepatic macrophages increased lipid accumulation in hepatocytes [5]. Stienstra et al. [6] demonstrated that hepatic macrophages promoted hepatic steatosis by suppressing peroxisome proliferator activated receptor alpha (Pparα) activity through IL1β-dependent mechanisms. Diehl et al. [7] demonstrated that the secretion of tumor necrosis factor (TNF) by hepatic macrophages was both necessary and sufficient to induce steatosis in hepatocytes *in vitro*. In methionine/choline deficient (MCD) diet-induced NASH model, hepatic macrophages exhibited an increased production of TNF, facilitated the infiltration of CD11b^int^Ly6C^hi^ monocytes, and triggered NASH development [8]. Zhang et al. [9] reported that macrophage p38α facilitated the progression of NASH through the induction of the cytokines secretion (TNF, C-x-c motif chemokine ligand 10 and interleukin 6) and pro-inflammatory macrophage polarization. Our previous research has indicated that the inhibition of Notch signaling in macrophages effectively suppressed the expression of inflammatory cytokines IL1β and TNF, thereby attenuating NASH in mice [10]. These findings suggest a strong association between the development of NAFLD and pro-inflammatory macrophages, emphasizing the potential significance of modulating the expression of key inflammatory factors, such as TNF or IL1β, in hepatic macrophages as a promising therapeutic strategy for treating NAFLD.

Exosomes, ranging from 30 to 150 nm in diameter, are phospholipid bilayer membrane vesicles secreted by living cells. Moreover, owing to their exceptional delivery efficiency and biocompatibility, exosomes hold great promise as a novel approach for drug delivery [11,12]. Importantly, circulating exogenous exosomes are primarily distributed in the liver and predominantly taken up by hepatic macrophages [10,[13], [14], [15]]. Therefore, exosomes represent natural delivery vehicles specifically targeting hepatic macrophages for the treatment of liver diseases.

In this study, we found that exosome-mediated delivery of antisense oligodeoxynucleotide against TNF (ASO-TNF) or 2-deoxy-d-glucose (2DG) effectively suppressed TNF and/or IL1β expression in macrophages. Furthermore, infusion of exosomes loaded with ASO-TNF or 2DG attenuated NASH progression by modulating the expression of superoxide dismutase 1 (Sod1) in mice.

## 2 Materials and methods

### 2.1 Antisense oligodeoxynucleotide against TNF (ASO-TNF)

Antisense oligodeoxynucleotide against TNF (ASO-TNF) have been reported in several references. [[16], [17], [18]] In brief, phosphorothioate-modified oligodeoxynucleotides (ODNs) were used, with cholesterol-labled at the 5′ terminus. In some localization studies, ASO-TNF were labeled with Texas-red dye at the 3′ terminus. The sequence of ASO-TNF (ISIS25302) was: 5′-AACCCATCGGCTGGCACCAC-3’; the sequence of the control antisense ODNs (ASO-CT) was 5′-TCAAGCAGTGCCACCGATCC-3’. The ODNs were synthesized by Tsingke Biotechnology (Beijing, China).

### 2.2 Isolation, identification and loading of exosomes

The mouse endothelial cell line bEnd.3 was cultured in Dulbecco's modified Eagle medium (DMEM) without serum medium for 48 h. Exosomes present in the supernatants were isolated using polyethylene glycol (PEG) 6000 (Sigma‐Aldrich, St. Louis, MO, USA) according to the established protocol [10,15]. Briefly, the culture medium was collected and sequentially centrifuged at 500×*g* for 5 min and 3000×*g* for 15 min to eliminate cells and cellular debris. The resulting supernatants were filtered through a 0.22-μm filter (Millipore, Billerica MA) and combined with a PEG6000 working solution to achieve a final concentration of 12 % PEG6000. The mixtures were then incubated at 4 °C for 12 h before being centrifuged at 12000×*g* for 60 min. Finally, the pellets were resuspended with PBS. The size distribution of exosomes was analyzed using nanoparticle tracking analysis (NTA) based on Laser Scattering Microscopy, while the morphology of exosomes was observed via transmission electron microscopy (TEM) at Dolaimi Biotechnology Co., Ltd. in Wuhan, China.

The exosomes (100 μg protein equivalent) were electroporated with 2DG (0.6 M, MedChemExpress, New Jersey, USA) using an electroporation system (Bio-Rad, California, USA) at 400 V and 125 μF in 0.4 cm electroporation cuvetes as previously described [10,15]. Alternatively, exosomes were incubated with cholesterol-modified ASO-TNF (Exo/ASO-TNF) or ASO-control (Exo/ASO-CT) for 30 min at 37 °C, using a concentration of 1.25 nmol ODNs per 100 μg exosomes. To remove any residual free 2DG or ODNs, the exosomes were washed and centrifuged with PBS containing 12 % PEG6000. The concentration of 2DG in exosomes (Exo/2DG) was determined using a glucose assay kit (Nanjing Jiancheng Bioengineering Institute, Nanjing, China) based on the glucose oxidase method. Given the comparable size of Biotin and 2DG, as well as the detectability advantage of Biotin, we evaluated the efficacy of electroporation for loading Biotin into exosomes. Exosomes (50 μg) was incubated in a 1 mM solution of Biotin. Following electroporation, the free Biotin was eliminated through washing and centrifugation. The concentration of Biotin in exosomes was detected by using fluorometric Biotin quantitation assay kit (Beyotime Biotechnology, Shanghai, China). To confirm the binding of cholesterol-modified ODNs and exosomes, we incubated exosomes with cholesterol-modified scrambled miRNAs (NC) or miR-188-5p mimics (miR), followed by qRT-PCR detection of the level of miR-188-5p in exosomes as previously described [15]. All the ODNs were synthesized by Tsingke Biotechnology. The sequences of ODNs are listed in Table S1.

### 2.3 Cell culture and treatment

Bone marrow-derived macrophages (BMDMs) were cultured according to the described protocol [15]. Mouse RAW264.7 macrophages were cultured in DMEM containing 10 % fetal bovine serum (FBS), 2 mM l-glutamine, 100 U mL^−1^ penicillin and 100 μg mL^−1^ streptomycin at 37 °C in a CO_2_ incubator maintained at a concentration of 5 %. To isolate primary hepatocytes and hepatic macrophages, mouse liver single-cell suspension was prepared by perfusion with collagenase IV (0.2 g L^−1^; Sigma). Subsequently, hepatocytes were harvested through centrifugation for 3 min at a speed of 50×*g*. Primary mouse hepatocytes were cultured using primary liver parenchyma cell culture system (iCell Bioscience, Shanghai, China). Hepatic macrophages were isolated utilizing magnetic bead cell sorting (MACS) as previously described [10,15]. The AML12 hepatocytes were cultured in special culture medium for AML12 cells (DMEM/F12 medium supplemented with 10 % FBS, 10 μg mL^−1^ insulin, 5.5 μg mL^−1^ transferrin, 5 ng mL^−1^ selenium, 40 ng mL^−1^ dexamethasone and antibiotics; Procell, Wuhan, China).

RAW264.7 cells or BMDMs were treated with 2DG (2 mM) and lipopolysaccharide (LPS, 200 ng mL^−1^, Sigma) for 24 h. Alternatively, RAW264.7 cells or BMDMs were transfected with ASO-TNF (100 nM) using lipofectamine 2000 (Thermo Fisher Scientific, Waltham, MA, USA) for 18–24 h, then stimulated with LPS for 24 h. For incubation with RAW264.7 cells or BMDMs, 20 μg protein equivalent of exosomes (Exo/ASO-CT, Exo/ASO-TNF, Exo or Exo/2DG) were added to 24-well plates and co-cultured for 24 h. Subsequently, the medium was replaced with fresh medium containing LPS (200 ng mL^−1^) or IL4 (10 ng mL^−1^, Pepro Tech, NJ, USA) and cultured for an additional 24 h. In certain experiments, the conditioned medium (CM) was collected.

For co-culture experiments, AML12 or primary hepatocytes were cultured with CM in palmitic acid medium (PA, 200 μM; Kunchuang Science and Technology Development Co., Ltd., Xi'an, China), with or without TNF (10 ng mL^−1^; CHAMOT Biotechnology, Shanghai, China) and/or IL-1β (10 ng mL^−1^; PrimeGene, Shanghai, China), for 48 h. In some cases, AML12 hepatocytes were transfected with Sod1 siRNA (50 nM; Tsingke Biotechnology), and 12 h later, cultured with CM and fresh palmitic acid medium (1:2) for 2 days. Hepatocytes were fixed in 4 % paraformaldehyde for 15 min and subsequently stained with freshly prepared saturated Oil red O solution (Servicebio, Wuhan, China) following established protocols. Triglyceride content was measured using a detection kit (Pulai Biological Co. LTD, Beijing) according to the manufacturer's recommended protocols.

### 2.4 Mice

C57BL/6 wild type mice were obtained from Gempharmatech Co., Ltd. (Nanjing, China). To induce experimental NASH, male mice (8 weeks old) were fed a choline deficient amino acid-defined diet (CDAA, l-amino acid diet with 45 % kcal% fat with 0.1 % methionine no added choline; Research Diets, New Brunswick, NJ, USA) for 10 weeks, or a methionine and choline deficient l-amino acid diet (MCD; Research Diets) for 4 weeks. All animal experiments were conducted in accordance with the guidelines set by the Animal Experiment Administration Committee of Fourth Military Medical University.

For exosome-mediated therapy, exosomes (Exo, Exo/ASO-TNF or Exo/2DG) were administered to CDAA-fed mice in the 9th and 10th week, or MCD-fed mice in the 3rd and 4th week via tail vein injection for four times (about 200 μg exsomes per mouse). In certain instances, Sod1 inhibitor LCS-1 (20 mg kg^−1^; MCE) dissolved in corn oil was injected intraperitoneally the next day after each exosome infusion. Mice were humanely euthanized for further analysis 2–3 days after the last exosome injection.

### 2.5 Histological analysis

The liver specimens were fixed with 4 % paraformaldehyde (PFA; Servicebio). Subsequently, PFA-fixed specimens were embedded in paraffin, sectioned. Hematoxylin and eosin (H&E) staining as well as Sirius red staining were performed following standard protocols.

For immunohistochemistry analysis, liver sections were prepared using established procedures. The primary antibodies used included anti-mouse F4/80 (Servicebio), Col1α1 (Servicebio) or αSMA (Servicebio). HRP-conjugated goat anti-rabbit IgG served as the secondary antibody (Servicebio). Antibody information is listed in Table S2.

For Oil Red O staining, PFA-fixed samples were embedded in optimal cutting temperature (OCT) compound and sectioned at a thickness of 8 μm. Subsequently, the sections were stained with freshly prepared saturated Oil Red O solution (Servicebio) following standard protocols. Immunofluorescence (IF) was performed as previously described [10,15]. Photomicrographs were captured using a microscope (Leica ICC50 HD, Wetzlar, Germany) or a fluorescence microscope (BX51, Olympus, Tokyo, Japan).

### 2.6 Biochemical analysis and detection of superoxide dismutase (SOD) activity

The levels of serum albumin, alanine aminotransferase (ALT), aspartate aminotransferase (AST), and triglyceride (TG) were quantified using an automatic biochemical analyzer (Rayto Life and Analytical Sciences Company, Shenzhen, China). The total activity of superoxide dismutase (SOD) in liver samples was determined using total SOD assay kit with WST-8 (Beyotime Biotechnology) following the manufacturer's instructions.

### 2.7 Western blot

Liver tissues, AML12 hepatocytes, RAW264.7 macrophages, bEnd.3 cells or exosomes were lysed using RIPA buffer (Beyotime) containing phenylmethylsulfonyl fluoride (PSMF, 1 mM). The protein concentration was quantified with the BCA Protein Assay Kit (Beyotime), following the manufacturer's instructions. Samples underwent sodium dodecyl sulfate-polyacrylamide gel electrophoresis (SDS-PAGE), transferred to polyvinylidene fluoride (PVDF) membranes (Millipore, Billerica MA), and incubated with primary antibodies targeting IL1β, TNFα, Tsg101, Alix, Flotillin-1,Vdac1, Pparγ, Sod1, Catalase, JNK, pJNK, Gapdh or ɑTubulin followed by HRP-conjugated goat anti-mouse or anti-rabbit IgG secondary antibodies. Antibody information is listed in Table S2.

### 2.8 RNA extraction and quantitative reverse transcription PCR (qRT-PCR)

Total RNA was extracted from liver tissues, RAW264.7 macrophages, BMDMs, hepatic macrophages, AML12 hepatocytes or exosomes using Trizol reagent (Invitrogen, Carlsbad, CA, USA) following the manufacturer's instructions. The mRNA was reverse transcribed into cDNA using Evo M-MLV RT Premix (Accurate Biology, Changsha, China). qRT-PCR was performed with SYBR Green Premix Pro Taq HS qPCR Kit (Accurate Biology) on a Bio-Rad CFX Maestro 2.2 Real-Time PCR System (Bio-Rad, Hercules, CA, USA). β-actin served as the internal control. Exosomal RNA was reverse transcribed into cDNA using Mir-X miRNA qRT-PCR SYBR kit (Takara, Dalian, China), with U6 as an internal control. All primers were purchased from Tsingke Biotechnology, and their sequences are listed in Table S1.

### 2.9 RNA-seq analysis

Liver samples were collected from Exo/ASO-TNF, Exo/2DG, or Exo treated CDAA-fed mice and subjected to RNA extraction followed by reverse transcription into cDNA for library construction. The cDNA libraries were sequenced on the Illumina sequencing platform by Genedenovo Biotechnology Co., Ltd (Guangzhou, China). The bioinformatic analysis was conducted by Genedenovo Biotechnology or through the utilization of a real-time interactive online platform Sangerbox (http://www.sangerbox.com/).

### 2.10 Statistical analysis

The histological images were imported into the Image Pro Plus 6.0 software (MediaCybernetics Inc., Bethesda, MD, USA) for analysis of the positive area. Western blot images were imported into Image J software and analyzed for integrated density. Data were analyzed using GraphPad Prism software, version 8.0. Comparisons between groups were performed using unpaired Student's t-test (two groups) or one-way ANOVA with Tukey's post hoc analysis (multiple groups). The number of repetitions is indicated in the legends of each graph. Results are presented as means ± SD with a significance level set at *P* < 0.05.

## 3 Results

### 3.1 TNF and IL1β were up-regulated in NASH patients or mouse NASH model, primarily originating from hepatic macrophages

**Fig. 1:**
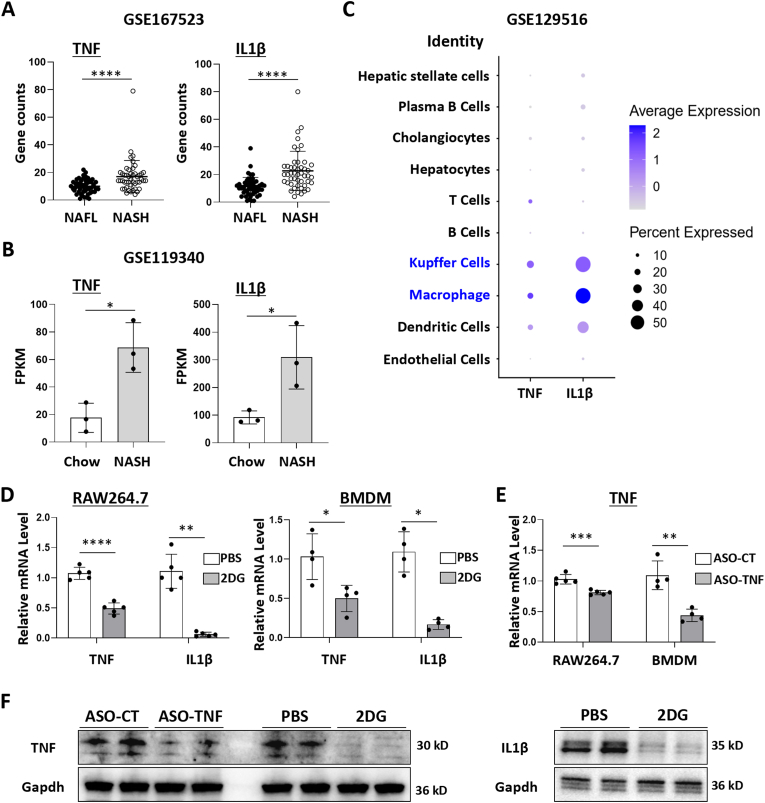
2-Deoxy-d-glucose (2DG) or antisense oligodeoxynucleotide against TNF (ASO-TNF) reduced the expression of inflammatory factors TNF and/or IL1β in macrophages. (A) Expression analysis of TNF and IL1β in human livers from transcriptome dataset GSE167523 in Gene Expression Omnibus (patients with NAFLD, NAFL = 51, NASH = 47). (B) Analysis of TNF and IL1β expression in the livers of chow-fed and NASH-induced mice from transcriptome dataset GSE119340. (C) Dot plot analysis was performed to assess the expression levels of TNF and IL1β in liver cell populations using scRNAseq dataset GSE129516. (D) RAW264.7 cells or BMDMs were treated with 2DG (2 mM) and lipopolysaccharide (LPS, 200 ng mL^−1^) for 24 h, and the expression of TNF and IL1β was detected using qRT-PCR. (E) RAW264.7 cells or BMDMs were transfected with ASO-TNF (100 nM) for 18–24 h, then stimulated with LPS for 24 h, and the expression of TNF was determined using qRT-PCR. (F) RAW264.7 cells were treated with 2DG or ASO-TNF, and the expression of TNF and/or IL1β was detected by Western blot, with Gapdh as a reference control. Bars = means ± SD, ∗*P* < 0.05, ∗∗*P* < 0.01, ∗∗∗*P* < 0.001, ∗∗∗∗*P* < 0.0001.

Analysis of the transcriptome dataset GSE167523 in Gene Expression Omnibus (consisting of NAFLD patients, with 51 cases of NAFL and 47 cases of NASH), revealed that the liver tissues from NASH patients exhibited higher expressions of TNF and IL1β compared to those from NAFL patients (Fig. 1A). Similarly, our analysis of transcriptome dataset GSE119340 from NASH-induced mice livers indicated that the expression levels of TNF and IL1β were elevated compared to those in the livers of chow-fed mice (Fig. 1B). Further, the expressions of TNF and IL1β in liver cell populations were evaluated using dot plot analysis of the scRNAseq dataset GSE129516, revealing hepatic macrophages (Kupffer and monocyte-derived macrophages) as the primary sources of TNF and IL1β (Fig. 1C).

### 3.2 2-Deoxy-d-glucose (2DG) or antisense oligodeoxynucleotide against TNF (ASO-TNF) reduced the expression of inflammatory factors TNF and/or IL1β in macrophages

Previous studies have demonstrated that pro-inflammatory macrophages predominantly rely on glycolysis for energy metabolism [[19], [20], [21]], and the classical inhibitor of the glycolytic pathway, 2-deoxy-d-glucose (2DG), can significantly suppress the expression of IL1β in macrophages [20]. Therefore, we selected 2DG as a candidate molecule to inhibit IL1β expression in macrophages. Meanwhile, considering the greater stability of DNA compared to RNA, we chose TNF antisense oligodeoxynucleotide (ISIS25302), which has been validated for its ability to inhibit TNF expression [[16], [17], [18]]. Initially, we assessed whether these two candidate molecules could inhibit IL1β or TNF expression *in vitro* using bone marrow-derived macrophages (BMDMs) or RAW264.7 cells. The mRNA and protein levels of TNF and IL1β were determined using quantitative reverse transcription PCR (qRT-PCR) and Western blot analysis after treatment with 2DG. Remarkably, 2DG not only effectively inhibited IL1β expression but also suppressed TNF expression at both transcriptional and translational levels (Fig. 1D–F). Additionally, BMDMs or RAW264.7 cells were transfected with ASO-TNF or ASO-control (ASO-CT). Subsequent qRT-PCR and Western blot analyses revealed that ASO-TNF efficiently attenuated TNF expression in macrophages (Fig. 1E and F). Thus, 2DG and ASO-TNF were identified as potential candidates for inhibiting IL1β and/or TNF expression in macrophages.

### 3.3 ASO-TNF or 2DG could be loaded with exosomes

**Fig. 2:**
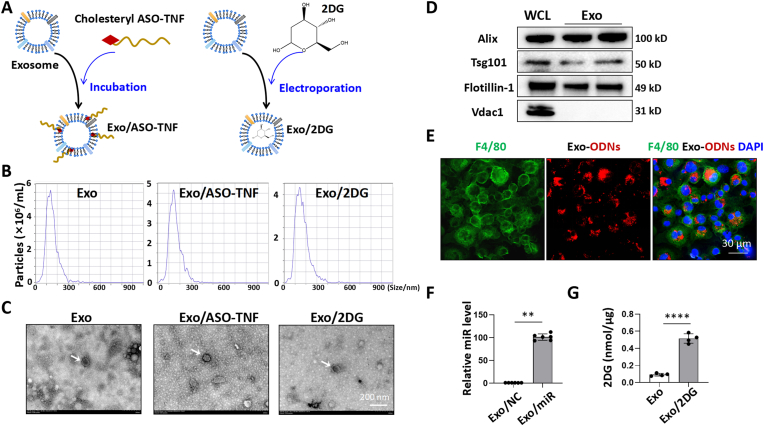
ASO-TNF or 2DG could be loaded with exosomes. (A) Schematic diagram of exosomes loaded with ASO-TNF or 2DG. (B) The size distribution of bEnd.3-derived exosomes was determined by nanoparticle tracking analysis (NTA). (C) Exosome morphology was observed by transmission electron microscopy (TEM). (D) The levels of Alix, Tsg101, Flotillin-1 and Vdac1 in bEnd.3 cells lysates and exosomes were detected by Western blot. (E) BMDMs were incubated with exosomes loaded with Texas-red labeled and cholesterol-modified oligonucleotides (1 μg exosomes/10 pmol ODNs). After 12 h, BMDMs were stained with an anti-mouse F4/80 antibody and analyzed by fluorescence microscopy. Nuclei were counterstained with DAPI. (F) Cholesterol-modified scrambled miRNAs (NC) or miR-188-5p mimics (miR) were incubated with exosomes and the level of miR-188-5p in exosomes were detected by qRT-PCR, with U6 as a reference control. (G) 2DG was loaded into exosomes by electroporation (0.6 M 2DG *VS.* 100 μg exosomes) and the concentration of 2DG in exosomes was measured by the glucose oxidase method. Bars = means ± SD; ∗∗*P* < 0.01, ∗∗∗∗*P* < 0.0001.

Exosomes injected intravenously were mainly distributed in the liver and taken up by hepatic macrophages [10,[13], [14], [15]]. This indicates that exosomes may represent a natural delivery system for the targeting of hepatic macrophages. In our previous work, we demonstrated that exosomes loaded with recombination signal binding protein for immunoglobulin kappa J region (RBP-J) decoy could inhibit Notch signaling in hepatic macrophages [10,15]. It is natural to ask whether ASO-TNF or 2DG could be loaded with exosomes and targeting inhibit TNF or IL1β expression in hepatic macrophages. As shown in Fig. 2A, the 5′ terminus of ASO-TNF was cholesterol-modified, and subsequently incorporated into exosomes’ membrane through its lipophilic properties following incubation with exosomes according to the methods in Refs. [[22], [23], [24]]. Additionally, electroporation was employed to load 2DG into exosomes. Due to its robust exosome production and minimal immunogenicity, we selected the mouse brain endothelial cell line 3 (bEnd.3) as our preferred cell line for exosome generation. Nanoparticle tracking analysis (NTA) assay revealed that the particle size of Exo, Exo/ASO-TNF or Exo/2DG remained comparable, with no significant impact observed due to incubation or electroporation (Fig. 2B). Transmission electron microscopy (TEM) analysis indicated that the exosomes maintained a bilayer structure and showed minimal alterations in morphology following incubation or electroporation (Fig. 2C). Furthermore, exosomes derived from bEnd.3 cells exhibited an enrichment of exosomal marker proteins Apoptosis-linked gene 2-interacting protein x (Alix), Tumor susceptibility 101 (Tsg101), and Flotillin-1, as confirmed by Western blot analysis. Conversely, the expression of mitochondrial membrane protein Voltage dependent anion channel 1 (Vdac1) was undetectable (Fig. 2D).

Exosomes were loaded with Texas red-labeled and cholesterol-modified oligodeoxynucleotides (ODNs), followed by a 12-h incubation with BMDMs. Fluorescence imaging revealed successful delivery of Texas red-labeled ODNs into macrophages by exosomes (Fig. 2E). In order to quantify ODNs within exosomes, we utilized cholesterol-modified miR-188-5p mimics for detection via qRT-PCR. Exosomes (10 μg protein) were incubated with either miR-188-5p mimics or negative control mimics (100 pmol). The results demonstrated a significant up-regulation of miR-188-5p levels in the loaded exosomes, approximately 100-fold higher compared to the control group (Fig. 2F). Next, we evaluated the efficiency of loading soluble small molecule 2DG into exosomes. Exosomes (100 μg) were incubated in a 600 mM solution of 2DG, and residual free 2DG was removed through washing and centrifugation after electroporation. The concentration of 2DG within exosomes was quantified using a glucose oxidase assay, indicating an average content of approximately 400 pmol μg^−1^ (Fig. 2G). Given the comparable size of Biotin and 2DG (molecular weights of 244 and 164, respectively), as well as the detectability advantage of Biotin, we assessed the effectiveness of electroporation in loading Biotin into exosomes. Exosomes (50 μg) was incubated in a 1 mM solution of Biotin. Following electroporation, the free Biotin was eliminated through washing and centrifugation. The concentration of Biotin in exosomes was detected by measuring the fluorescence intensity emitted by fluorescein isothiocyanate (FITC)-labeled Streptavidin, which revealed a concentration of approximately 1 pmol μg^−1^ (Fig. S1, Supporting Information). These results indicated that water-soluble small molecules, such as 2DG and Biotin were efficiently loaded via electroporation. These findings provide confirmation that ASO-TNF or 2DG can be effectively loaded with exosomes.

### 3.4 Exo/ASO-TNF or Exo/2DG inhibited TNF and/or IL1β expression in macrophages *in vitro* and *in vivo*

**Fig. 3:**
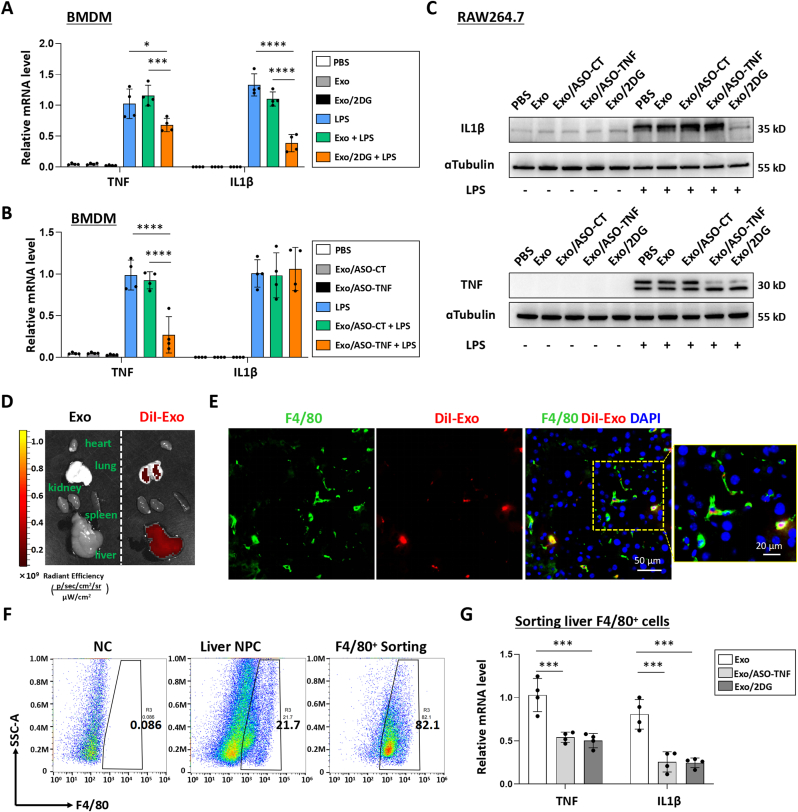
Exo/ASO-TNF or Exo/2DG inhibited TNF and/or IL1β expression in macrophages *in vitro* and *in vivo*. (A-B) BMDMs were incubated with Exo, Exo/2DG, Exo/ASO-Control (Exo/ASO-CT) or Exo/ASO-TNF for 24 h, then changed and cultured with the fresh medium containing LPS (200 ng mL^−1^) for another 24 h, and the expression of TNF and IL1β was detected by qRT-PCR. (C) RAW264.7 macrophages were incubated with Exo, Exo/2DG, Exo/ASO-CT or Exo/ASO-TNF for 24 h, then treated with LPS (200 ng mL^−1^) for 24 h, and the expression of TNF and IL1β was detected by Western blot, with ɑTubulin as a reference control. (D) DiI-labled exosomes (200 μg) were injected via the tail vein into MCD-fed mice. After 6 h, DiI signals in the liver, lung, spleen, kidney, and heart were examined using bioluminescence imaging. (E) Liver sections were stained with an anti-mouse F4/80 antibody and analyzed by fluorescence microscopy. Nuclei were counterstained with DAPI. (F) Mice were fed with MCD diet for 4 weeks, and Exo/ASO-TNF or Exo/2DG (200 μg) were injected into mice four times via tail vein at the last 2 weeks. F4/80^+^ hepatic macrophages were isolated using magnetic-activated cell sorting (MACS). The purity of F4/80^+^ cells were determined by flow cytometry. (G) The mRNA levels of TNF and IL1β in F4/80^+^ hepatic macrophages were measured by qRT-PCR. Bars = means ± SD; ∗*P* < 0.05, ∗∗*P* < 0.01, ∗∗∗*P* < 0.001, ∗∗∗∗*P* < 0.0001.

The potential functionality of Exo/ASO-TNF or Exo/2DG in macrophages was subsequently assessed. BMDMs or RAW264.7 cells were co-cultured with Exo, Exo/2DG, Exo/ASO-CT or Exo/ASO-TNF for 24 h, and then stimulated with lipopolysaccharide (LPS, 200 ng mL^−1^) for an additional 24 h. The expression levels of TNF and IL1β was detected by using qRT-PCR and Western blot analysis. As shown in Fig. 3A–C, treatment with Exo/ASO-TNF resulted in a reduction in both mRNA and protein levels of TNF in macrophages, while treatment with Exo/2DG exhibited inhibitory effects on the expression of both TNF and IL1β in macrophages. Meanwhile, the expression of macrophage M1 polarization-related markers, including inducible nitric oxide synthase (iNOS), interleukin 6 (IL6) and interleukin 12 (IL12) was evaluated. As depicted in Fig. S2A, Exo/ASO-TNF did not exert a significant impact on the expression of these genes; however, Exo/2DG could down-regulated the expression of IL12 and up-regulated the expression of iNOS and IL6. Additionally, the effects of Exo/ASO-TNF or Exo/2DG on macrophage M2 polarization were also assessed. The results indicated that Exo/2DG could promote the expression of arginase1 (Arg1), Cluster of Differentiation 163 (Cd163) and interleukin 10 (IL10), whereas Exo/ASO-TNF had no significant effect on these M2 polarization-related genes (Fig. S2B).

Subsequently, the distribution of intravenously injected exosomes in mice with steatohepatitis was examined. A mouse model of steatohepatitis was established by administering an methionine and choline deficient (MCD) diet. DiI-labeled exosomes (200 μg per mouse) were administered via the tail vein. Consistent with our previous studies [10,15], bioluminescence imaging analysis revealed that DiI-labeled exosomes predominantly accumulated in the liver 6 h post-injection (Fig. 3D). Furthermore, immunofluorescence staining of liver sections demonstrated that DiI-labeled exosomes primarily localized within F4/80^+^ macrophages (Fig. 3E). Then, MCD-fed mice received infusion of Exo, Exo/ASO-TNF or Exo/2DG. Hepatic F4/80^+^ cells were isolated using magnetic activated cell sorting (MACS) as previous reported [10,15], achieving a purity level of 82 % (Fig. 3F). As determined by qRT-PCR, the expression levels of TNF and IL1β were down-regulated in hepatic F4/80^+^ cells derived from mice treated with Exo/ASO-TNF or Exo/2DG compared to those treated with Exo control (Fig. 3G). Exo/ASO-TNF may exert an indirect inhibitory effect on the expression of IL1β *in vivo*. These results suggested that Exo/ASO-TNF or Exo/2DG have the potential to inhibit TNF and/or IL1β expression in macrophages *in vitro* and *in vivo*.

### 3.5 Exo/ASO-TNF or Exo/2DG attenuated lipid accumulation in hepatocytes *in vitro*

**Fig. 4:**
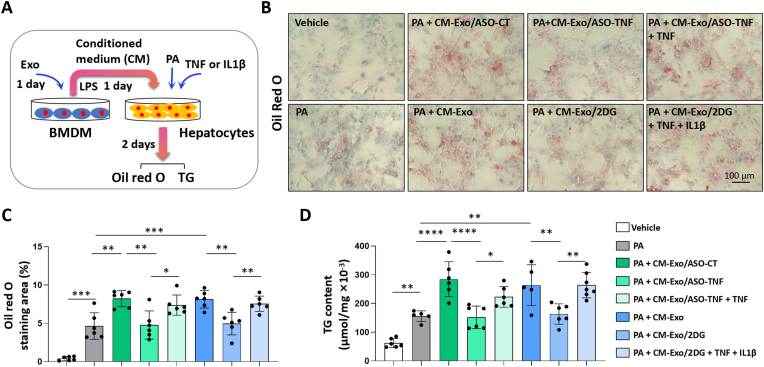
Exo/ASO-TNF or Exo/2DG attenuated lipid accumulation in primary hepatocytes *in vitro*. (A) Schematic showing that BMDMs were treated with Exo/ASO-TNF or Exo/2DG for 1 day, changed and cultured with the fresh medium containing LPS for 1 day, and the conditioned medium (CM) were harvested. Then primary mouse hepatocytes were cultured with CM, in the presence or absence of TNF (10 ng mL^−1^), and/or IL1β (10 ng mL^−1^) in palmitic acid medium (PA, 200 μM) for 2 days. (B) Lipid accumulation in primary hepatocytes was assessed using Oil Red O staining. (C) The positive areas of Oil Red O staining in (B) were quantitatively compared. (D) Triglyceride (TG) content in primary hepatocytes was measured. Bars = means ± SD, ∗*P* < 0.05, ∗∗*P* < 0.01, ∗∗∗*P* < 0.001, ∗∗∗∗*P* < 0.0001.

Previous studies have demonstrated that the pro-inflammatory cytokines IL1β and TNF can promote lipid accumulation in hepatocytes [[5], [6], [7], [8]]. In this study, we asked whether treatment with Exo/ASO-TNF or Exo/2DG could impair lipid accumulation in hepatocytes. As shown in Fig. 4A and Fig. S3A, BMDMs or RAW264.7 macrophages were treated with Exo, Exo/ASO-TNF or Exo/2DG for 24 h, followed by incubation with LPS-containing medium for another 24 h to obtain conditioned medium (CM). Primary hepatocytes or AML12 cells were then cultured with CM supplemented with or without TNF/IL1β for 48 h, and lipid accumulation was assessed using Oil Red O staining and triglyceride content detection. Our results showed that CM derived from treatment with either Exo/ASO-TNF or Exo/2DG significantly attenuated the capacity to induce lipid accumulation in primary hepatocytes or AML12 cells compared to control CM. Moreover, supplementation of TNF and/or IL1β partially restored this effect (Fig. 4B–D and Fig. S3B-D).

### 3.6 Exo/ASO-TNF or Exo/2DG attenuated experimental steatohepatitis in CDAA-fed or MCD-fed mice

**Fig. 5:**
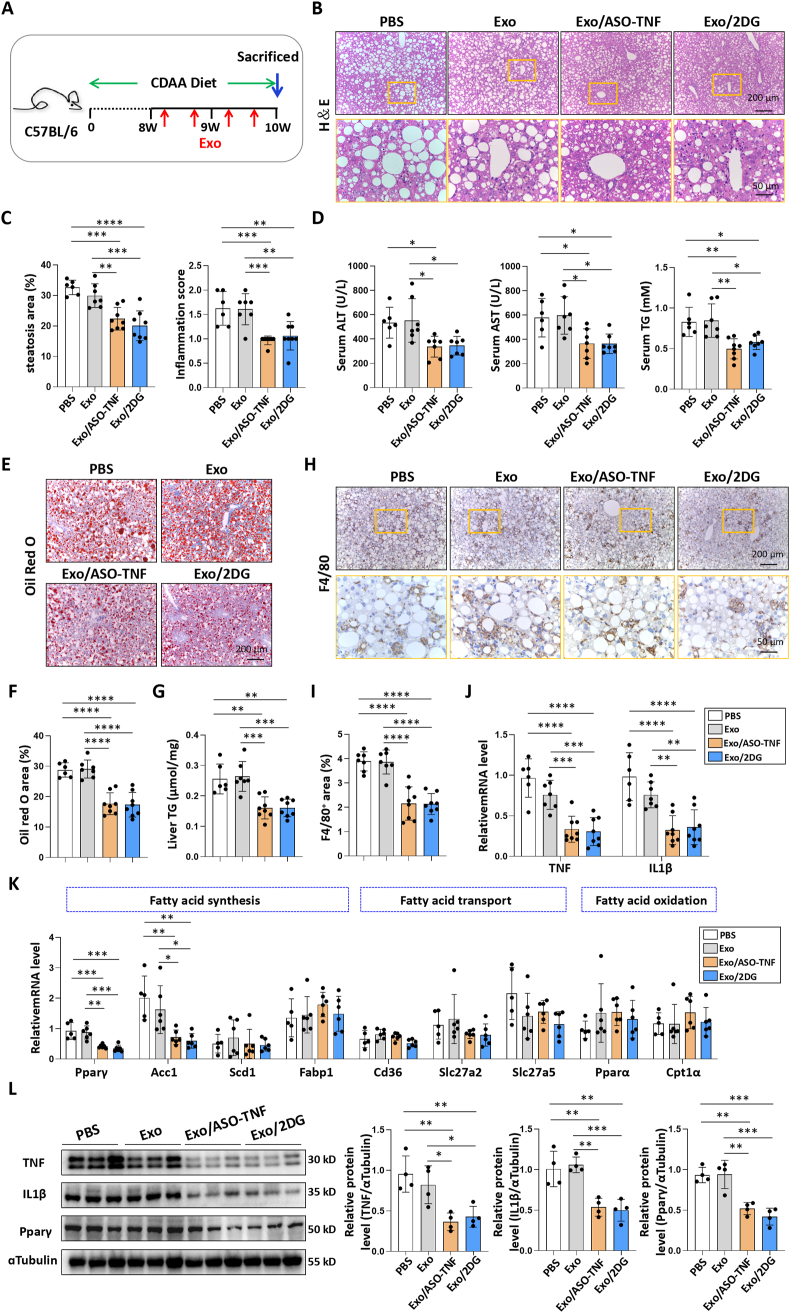
Exo/ASO-TNF or Exo/2DG attenuated experimental steatohepatitis in CDAA-fed mice. (A) Schematic illustration of the procedure used for Exo/ASO-TNF or Exo/2DG to treat steatohepatitis in mice fed with CDAA diet. Mice were fed with CDAA diet for 10 weeks, and Exo/ASO-TNF or Exo/2DG (200 μg) were injected into mice four times via tail vein at the last 2 weeks. (B) Liver sections were stained by H&E staining. The lower row of micrographs were a higher magnification of the yellow frames in the upper row. (C) Quantitative comparison of steatosis areas and inflammation scores in (B). (D) Detection of ALT, AST and TG in serum. (E,F) Liver sections were stained with Oil Red O, and positive areas were quantified and compared. (G) Detection of TG in liver tissue homogenate. (H,I) Liver sections were subjected to immunohistochemical staining for F4/80. The positive areas of F4/80 staining were quantitatively compared (I). (J-K) The mRNA levels of TNF, IL1β and fatty acid metabolism-associated genes in livers were determined by qRT-PCR. (L) The protein levels of TNF, IL1β and Pparγ were determined by Western blot, with ɑTubulin as a reference control. The integrated density were quantitatively compared. Bars = means ± SD, ∗*P* < 0.05, ∗∗*P* < 0.01, ∗∗∗*P* < 0.001, ∗∗∗∗*P* < 0.0001.

**Fig. 6:**
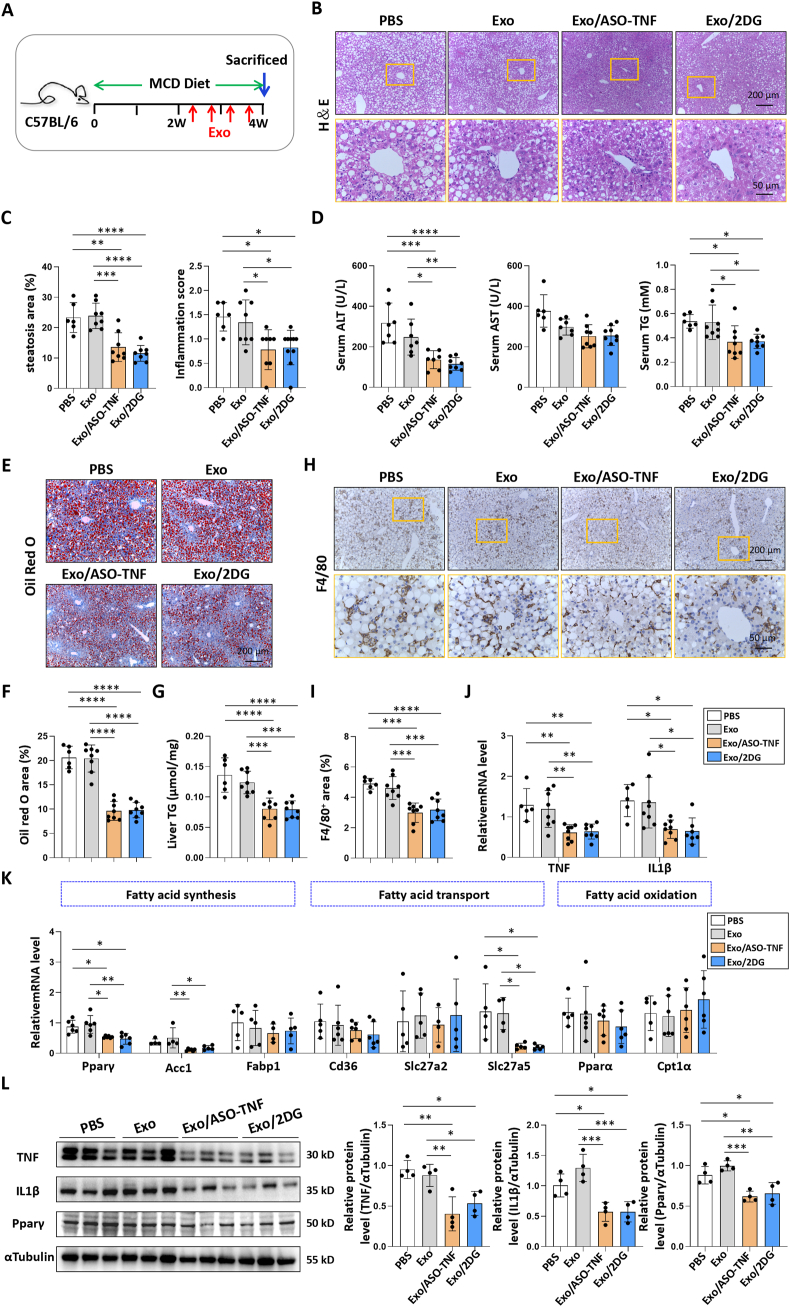
Exo/ASO-TNF or Exo/2DG attenuated experimental steatohepatitis in MCD-fed mice. (A) Schematic illustration of the procedure used for Exo/ASO-TNF or Exo/2DG to treat steatohepatitis in mice fed with MCD diet. (B) Liver sections were stained by H&E staining. The lower row of micrographs were a higher magnification of the yellow frames in the upper row. (C) Quantitative comparison of steatosis areas and inflammation scores in (B). (D) Detection of ALT, AST and TG in serum. (E,F) Liver sections were stained with Oil Red O, and staining positive areas were quantified and compared. (G) Detection of TG in liver tissue homogenate. (H,I) Liver sections were subjected to immunohistochemical staining for F4/80. The lower row of micrographs were a higher magnification of the yellow frames in the upper row. The positive areas of F4/80 staining were quantitatively compared (I). (J-K) The mRNA levels of TNF, IL1β and fatty acid metabolism-associated genes in livers were determined by qRT-PCR. (L) The protein levels of TNF, IL1β and Pparγ in livers were determined by Western blot, with ɑTubulin as a reference control. The integrated density were quantitatively compared. Bars = means ± SD, ∗*P* < 0.05, ∗∗*P* < 0.01, ∗∗∗*P* < 0.001, ∗∗∗∗*P* < 0.0001.

Next, we evaluated the therapeutic efficacy of Exo/ASO-TNF or Exo/2DG in choline deficient amino acid-defined diet (CDAA)-fed or MCD-fed mice. As shown in Fig. 5A and 6A, the mice were subjected to a 10-week CDAA diet or a 4-week MCD diet, followed by four intravenous infusions of Exo, Exo/ASO-TNF, or Exo/2DG (200 μg per mouse) during the final 2 weeks. Compared to control mice, administration of either Exo/ASO-TNF or Exo/2DG attenuated serum alanine aminotransferase (ALT) and aspartate aminotransferase (AST) levels, indicating amelioration of hepatic injury in treated mice (Fig. 5D; Fig. 6D). Hematoxylin and eosin (H&E) staining revealed fewer large round non-staining areas, indicative of lipid droplets in hepatocytes, in livers from mice treated with Exo/ASO-TNF or Exo/2DG compared to control mice (Fig. 5B and C; Fig. 6B and C). Oil Red O staining of liver sections showed a decreased presence of lipid droplets in the Exo/ASO-TNF or Exo/2DG treated group (Fig. 5E and F; Fig. 6E and F). Consistent with these findings, both serum and liver triglyceride levels exhibited significant reductions in mice treated with Exo/ASO-TNF or Exo/2DG (Fig. 5D,G; Fig. 6D,G). The expression of genes involved in lipid metabolism in the liver, including fatty acid synthesis, transport and oxidation, was detected by qRT-PCR. Our results showed that treatment with Exo/ASO-TNF or Exo/2DG mainly down-regulated the expression of key genes associated with fatty acid synthesis, such as the transcription factor peroxisome proliferator activated receptor gamma (Pparγ) and the enzyme acetyl-CoA carboxylase1 (Acc1)(Fig. 5K; Fig. 6K). Furthermore, Western blot analysis confirmed a decrease in Pparγ expression in livers treated with Exo/ASO-TNF or Exo/2DG (Fig. 5L; Fig. 6L). These results suggested that infusion of Exo/ASO-TNF or Exo/2DG could effectively mitigate hepatic lipid accumulation.

Meanwhile, liver inflammation and fibrosis were assessed. Firstly, our results showed that infusion of Exo/ASO-TNF or Exo/2DG resulted in a reduction in the inflammation score (Fig. 5C; Fig. 6C). Secondly, immunostaining with anti-F4/80 revealed decreased macrophage infiltration in livers from mice treated with Exo/ASO-TNF or Exo/2DG (Fig. 5H and I; Fig. 6H and I). Furthermore, the levels of inflammatory mediators TNF and IL1β were determined using qRT-PCR and Western blot. The results indicated that TNF and IL1β were decreased in livers from mice treated with Exo/ASO-TNF or Exo/2DG compared to control mice (Fig. 5J,L; Fig. 6J,L). These findings suggested that the infusion of Exo/ASO-TNF or Exo/2DG attenuated hepatic inflammatory response. Subsequently, we evaluated hepatic fibrosis by performing immunostaining for anti-collagen type I alpha 1 chain (Col1α1), anti-actin alpha 2 smooth muscle (αSMA), and Sirius red staining. Mice treated with Exo/ASO-TNF or Exo/2DG displayed significantly reduced extracellular matrix (ECM) deposition and activation of hepatic stellate cells (Fig. S4). Over all, these results indicated that Exo/ASO-TNF or Exo/2DG could ameliorate CDAA- or MCD diet-induced NASH in mice.

### 3.7 Exo/ASO-TNF or Exo/2DG could attenuate lipid accumulation in hepatocytes via Sod1

**Fig. 7:**
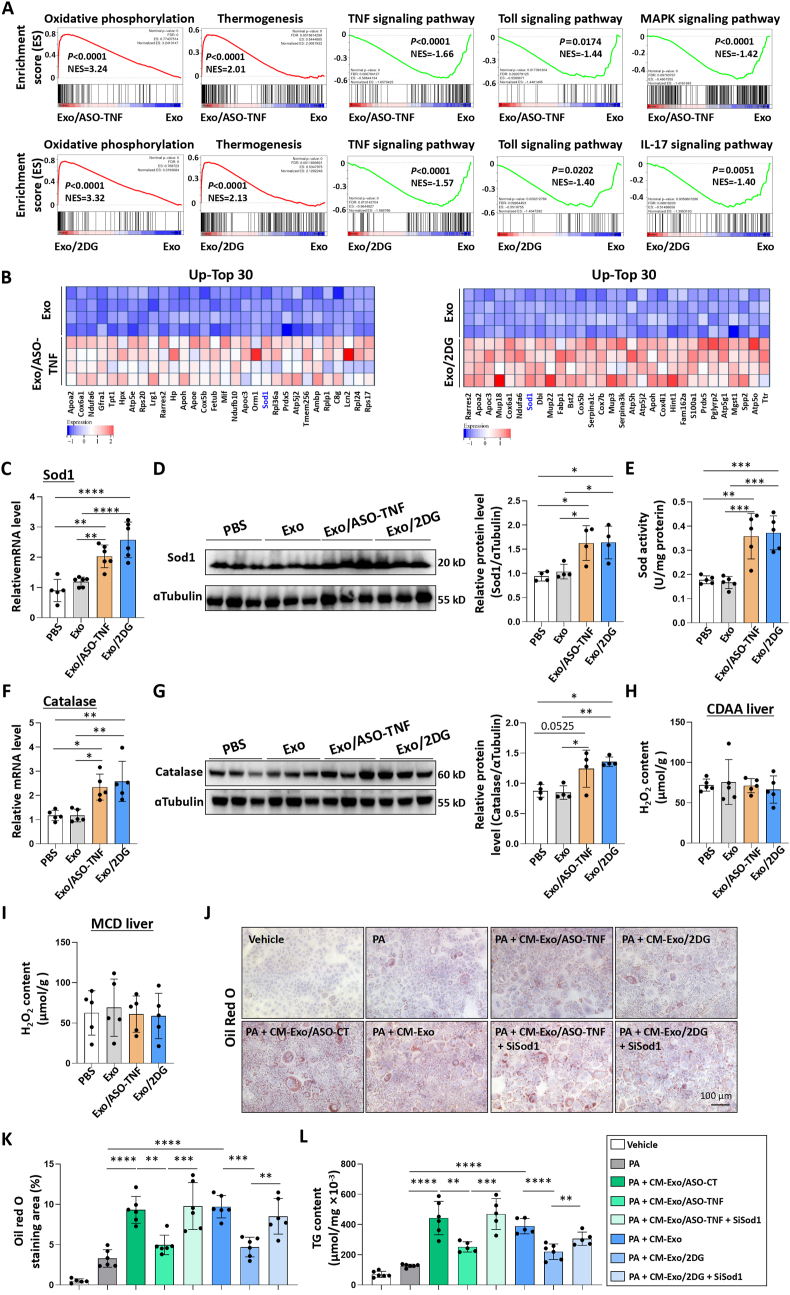
Exo/ASO-TNF or Exo/2DG could attenuate lipid accumulation in hepatocytes via Sod1. (A) The mRNA expression of liver samples from Exo/ASO-TNF, Exo/2DG or Exo-treated CDAA-fed mice was profiled by using RNA-seq (n = 4). Gene set enrichment analyses between Exo/ASO-TNF or Exo/2DG and Exo groups were investigated. (B) Heatmaps showed top 30 up-regulated genes in the RNA-seq data between Exo/ASO-TNF, or Exo/2DG and Exo groups. (C) The mRNA level of Sod1 in livers of PBS, Exo, Exo/ASO-TNF or Exo/2DG-treated mice was measured by qRT-PCR. (D) The level of Sod1 in livers of PBS, Exo, Exo/ASO-TNF or Exo/2DG-treated mice was detected by Western blot. The integrated density were quantitatively compared. (E) Detection of total Sod activity in liver tissue homogenate. (F) The mRNA level of Catalase in livers of PBS, Exo, Exo/ASO-TNF or Exo/2DG-treated CDAA-fed mice was measured by qRT-PCR. (G) The level of Catalase in livers of PBS, Exo, Exo/ASO-TNF or Exo/2DG-treated CDAA-fed mice was detected by Western blot. The integrated density were quantitatively compared. (H,I) Detection of H_2_O_2_ content in liver tissue obtained from CDAA-mice or MCD-mice. (J) AML 12 hepatocytes were transfected with Sod1 siRNA, and 12 h later, cultured with RAW264.7 conditioned medium (CM) and fresh palmitic acid medium for 2 days. Lipid accumulation in AML12 cells was assessed using Oil Red O staining. (K) The positive areas of Oil Red O staining in (J) were quantitatively compared. (L) Triglyceride (TG) content in AML12 hepatocytes was measured. Bars = means ± SD, ∗*P* < 0.05, ∗∗*P* < 0.01, ∗∗∗*P* < 0.001, ∗∗∗∗*P* < 0.0001.

To elucidate the underlying mechanism by which infusion of Exo/ASO-TNF or Exo/2DG mitigated experimental NASH in mice, we performed RNA-seq analysis to compare hepatic mRNA profiles between mice treated with Exo/ASO-TNF or Exo/2DG and Exo control. Gene set enrichment analysis showed an up-regulation of oxidative phosphorylation and thermogenesis associated genes, while a down-regulation was observed in inflammatory signaling pathways such as TNF, Toll, Mitogen-activated protein kinase (MAPK) or interleukin 17 (IL17) signaling pathway in mice treated with Exo/ASO-TNF or Exo/2DG (Fig. 7A). The qRT-PCR results confirmed that treatment with Exo/ASO-TNF or Exo/2DG resulted in the up-regulation of Apolipoprotein a2 (Apoa2, whose defects may result in hypercholesterolemia [25]), NADH:ubiquinone oxidoreductase subunit a6 (Ndufa6, associated with mitochondrial respiratory chain) and Cytochrome C oxidase subunit 6a1 (Cox6a1, associated with mitochondrial respiratory chain) in the liver (Fig. S5C). Additionally, Exo/ASO-TNF or Exo/2DG reduced the expression of inflammatory genes such as intercellular adhesion molecule 1 (Icam1) and interleukin 1 receptor associated kinase 1 (Irak1)(Fig. S5B and S5C).

Superoxide dismutase 1 (Sod1) caught our attention as one of the up-regulated top 30 genes in both Exo/ASO-TNF and Exo/2DG treated groups (Fig. 7B). Previous studies have reported the robust expression of Sod1 in hepatocytes, and genetic ablation of Sod1 has been shown to exacerbate hepatic lipid accumulation [[26], [27], [28]]. We asked whether Exo/ASO-TNF or Exo/2DG treatment enhanced the expression of Sod1 in the liver, thereby mitigating experimental NASH. Initially, we validated the up-regulation of Sod1 expression in livers following Exo/ASO-TNF or Exo/2DG treatment using qRT-PCR and Western blot analysis (Fig. 7C and D). Subsequently, we quantified the overall hepatic Sod activity and observed that Exo/ASO-TNF or Exo/2DG treatments increased hepatic Sod activity compared to the control group treated with Exo (Fig. 7E). Sod1 facilitates the conversion of superoxide anion into hydrogen peroxide, which is subsequently decomposed into oxygen and water by Catalase [29]. Therefore, hepatic hydrogen peroxide content was measured, and the results demonstrated that the H_2_O_2_ levels in the liver of both Exo/ASO-TNF or Exo/2DG-treated groups were comparable to those in the control group (Fig. 7H–I). We then assessed the expression of Catalase and observed that Exo/ASO-TNF or Exo/2DG treatment significantly up-regulated both mRNA and protein levels of Catalase in the livers of CDAA-fed mice (Fig. 7F–G). This may explain why increased Sod1 expression did not result in a significant increase of H_2_O_2_ levels.

Next, we performed an *in vitro* rescue experiment by silencing the expression of Sod1 in AML12 hepatocytes using siRNA, which was confirmed by qRT-PCR and Western blot (Fig. S6A and S6B). The transfected AML12 hepatocytes were then cultured with CM derived from RAW264.7 cells treated with Exo, Exo/ASO-TNF or Exo/2DG. Lipid accumulation was assessed using Oil Red O staining and triglyceride content detection (Fig. S6C). Our results indicated that CM treated with either Exo/ASO-TNF or Exo/2DG reduced the ability to induce lipid accumulation in AML12 hepatocytes compared to control CM, while knockdown of Sod1 in AML12 cells nearly restored this effect (Fig. 7J–L). These results suggested that Exo/ASO-TNF or Exo/2DG could reduce lipid accumulation in hepatocytes by up-regulating Sod1 expression.

### 3.8 Sod1 inhibitor LCS-1 counteracted the therapeutic effects of Exo/ASO-TNF or Exo/2DG on CDAA-induced NASH in mice

**Fig. 8:**
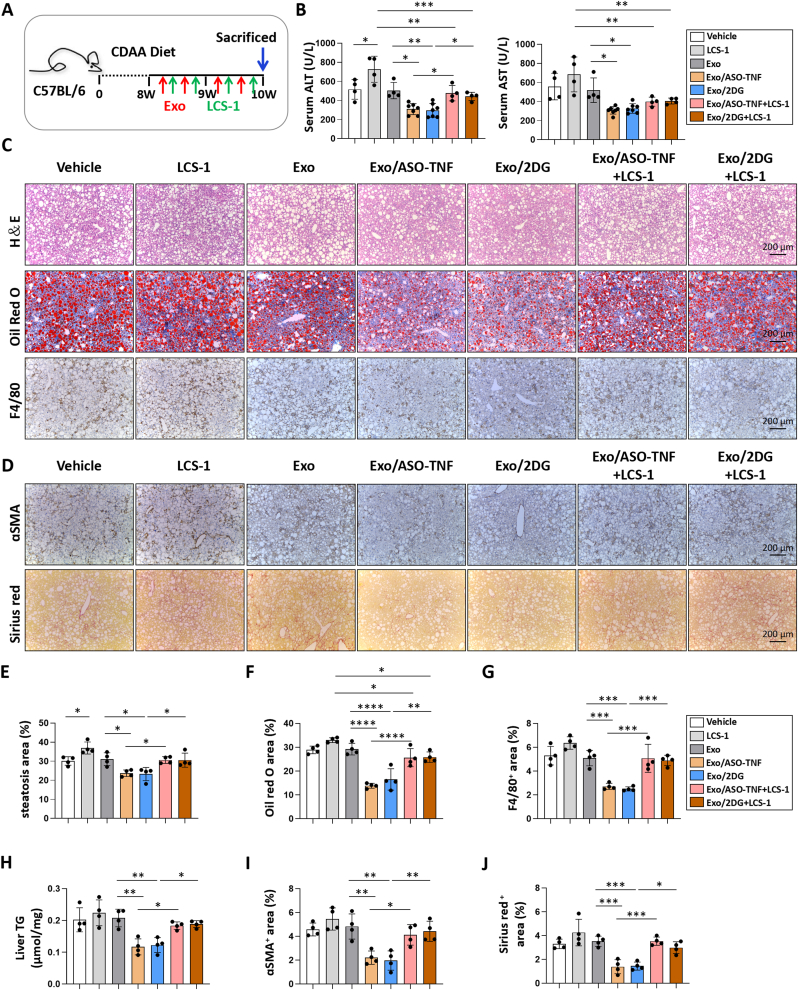
Sod1 inhibitor LCS-1 counteracted the therapeutic effects of Exo/ASO-TNF or Exo/2DG on CDAA-induced NASH in mice. (A) Schematic illustration of the procedure used for Exo/ASO-TNF or Exo/2DG and Sod1 inhibitor LCS-1 to treat steatohepatitis in mice fed with CDAA diet. Exo/ASO-TNF or Exo/2DG (200 μg) were injected into mice four times via tail vein at the last 2 weeks. LCS-1 (20 mg kg^−1^) dissolved in corn oil was injected intraperitoneally the next day after each exosome infusion. (B) Detection of ALT and AST in serum. (C) Liver sections of CDAA-fed mice were subjected to H&E staining, Oil Red O staining, or immunohistochemical staining for F4/80. (D) Liver sections were subjected to Sirius Red staining, or immunohistochemistry with anti-αSMA. (E-G) Quantitative comparison of steatosis areas, Oil Red O staining positive areas or F4/80^+^ areas in (C). (H) Detection of TG in liver tissue homogenate. (I,J) Positive signals for immunohistochemistry staining of αSMA or Sirius Red staining in (D) were quantitatively compared. Bars = means ± SD, ∗*P* < 0.05, ∗∗*P* < 0.01, ∗∗∗*P* < 0.001, ∗∗∗∗*P* < 0.0001.

We further performed an *in vivo* rescue experiment. As shown in Fig. 8A, the Sod1-specific inhibitor LCS-1 was intraperitoneally injected one day after each infusion of Exo/ASO-TNF or Exo/2DG in CDAA diet-induced NASH model. It was observed that LCS-1 elevated the levels of ALT in serum of mice treated with Exo/ASO-TNF or Exo/2DG (Fig. 8B). Histological analysis using H&E staining, Oil Red O staining, and liver TG content detection revealed that LCS-1 counteracted the impact of Exo/ASO-TNF or Exo/2DG on hepatic lipid accumulation (Fig. 8C,E,F,H). Immunostaining for anti-F4/80 demonstrated that LCS-1 increased macrophage infiltration in the livers of mice treated with Exo/ASO-TNF or Exo/2DG (Fig. 8C,G). Immunostaining for anti-ɑSMA and Sirius red staining indicated that LCS-1 reversed the inhibitory effect of Exo/ASO-TNF or Exo/2DG on liver fibrosis (Fig. 8D,I,J). These findings suggested that Exo/ASO-TNF or Exo/2DG could inhibit NASH progression through up-regulation of Sod1 in CDAA-fed mice model.

### 3.9 Sod1 was negatively correlated with JNK activation in hepatocytes treated with TNF or livers of CDAA-fed mice

**Fig. 9:**
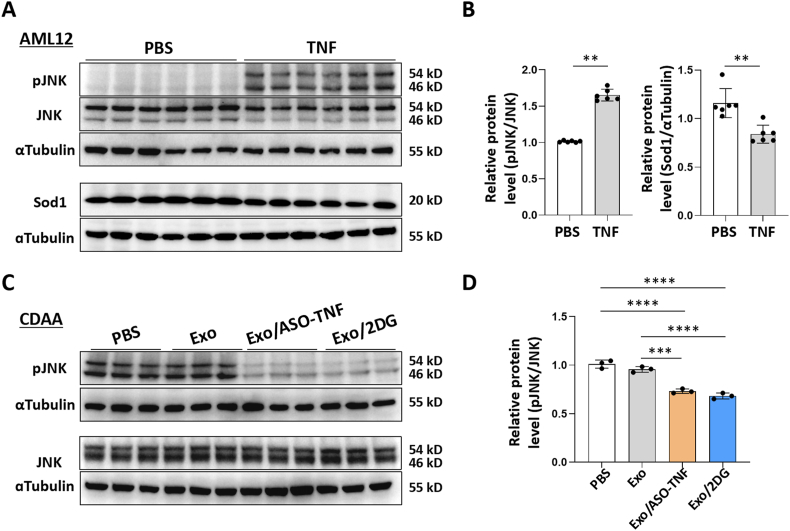
The expression of Sod1 was negatively correlated with JNK activation in hepatocytes treated with TNF or livers of CDAA-fed mice. (A, B) AML12 hepatocytes were treated with TNF (10 ng ml^−1^) or PBS in palmitic acid medium (PA, 200 μM) for 2 days. Subsequently, the protein levels of pJNK, JNK, Sod1 and ɑTubulin were determined by Western blot, and the integrated density of pJNK or Sod1 was quantitatively compared. (C, D) The protein levels of pJNK, JNK and ɑTubulin in livers of CDAA-fed mice were assessed using Western blot analysis. Subsequently, the integrated density was quantitatively compared. Bars = means ± SD, ∗*P* < 0.05, ∗∗*P* < 0.01, ∗∗∗*P* < 0.001, ∗∗∗∗*P* < 0.0001.

How did Exo/ASO-TNF or Exo/2DG modulate the expression of Sod1 in hepatocytes? Previous studies have demonstrated that TNF can suppress Sod1 expression through JNK pathway [30]. We then stimulated AML12 cells with TNF and observed an increase of pJNK levels, along with a simultaneous decrease of Sod1 expression (Fig. 9A and B). Additionally, we assessed JNK signaling activation in livers from CDAA-fed mice and found that treatment with Exo/ASO-TNF or Exo/2DG significantly reduced pJNK levels, indicating their inhibitory effect on JNK pathway activation (Fig. 9C and D). Based on these results, we speculated that Exo/ASO-TNF or Exo/2DG may down-regulate inflammatory factors such as TNF, thereby attenuating JNK signaling activation in hepatocytes and subsequently promoting the up-regulation of Sod1 expression.

### 3.10 Exo/ASO-TNF or Exo/2DG decreased the level of reactive oxygen species (ROS) in hepatocytes via Sod1

Sod1 is one of the important antioxidant enzymes that metabolizes superoxide radicals to molecular oxygen and hydrogen peroxide [29,31,32]. Therefore, we examined the effects of Exo/ASO-TNF or Exo/2DG on reactive oxygen species (ROS). Primary hepatocytes were isolated from livers of MCD-fed mice, and total ROS were detected using 2′,7′-Dichlorofluorescin diacetate (DCFH-DA) staining. The results showed that treatment with Exo/ASO-TNF or Exo/2DG significantly decreased the total ROS level in primary hepatocytes (Fig. S7A). Moreover, AML12 hepatocytes were co-cultured with conditioned medium derived from RAW264.7 cells treated with Exo, Exo/ASO-TNF or Exo/2DG. Because DCFH-DA staining was too weak in cultured AML12 cells (data not shown), and since mitochondria are the main source of ROS, mitochondrial superoxides were assessed using MitoSOX red staining. The results indicated that both Exo/ASO-TNF and Exo/2DG effectively attenuated the levels of mitochondrial superoxides in hepatocytes as determined by FACS (Fig. S7B) and fluorescence imaging (Fig. S7D-E). Meanwhile, we quantified the adenosine 5′-triphosphate (ATP) content in AML12 hepatocytes, which serves as an indicator of mitochondrial function. The results revealed that both Exo/ASO-TNF and Exo/2DG treatments enhanced ATP production in AML12 cells compared to the control group (Fig. S7C). Interestingly, we performed Gene Ontology analysis of hepatic RNA-seq data focusing on the mitochondrial respiratory chain complex (GO: 0098803) and observed a significant up-regulation of genes associated with this complex following treatment with either Exo/ASO-TNF or Exo/2DG (Fig. S5D). These findings indicated an improvement in mitochondrial function of hepatocytes upon exposure to Exo/ASO-TNF or Exo/2DG. Furthermore, we noted that the reduction in ROS levels induced by either Exo/ASO-TNF or Exo/2DG was counteracted by LCS-1, an inhibitor of Sod1 activity (Fig. S7D-E). Additionally, treatment with either Exo/ASO-TNF or Exo/2DG resulted in a decrease in superoxide anion content within AML12 hepatocytes, which could also be reversed by LCS-1 administration (Fig. S7F).

## 4 Discussion

NASH is characterized by hepatic steatosis, liver inflammation and hepatic fibrosis. Inflammation plays a crucial role in linking hepatic steatosis to the progression of NASH towards hepatic fibrosis or cirrhosis [[2], [3], [4]]. A comprehensive understanding of inflammation is crucial for elucidating the underlying mechanisms driving NASH and developing targeted therapeutic interventions capable of halting or reversing its progression. The pro-inflammatory cytokines TNF and IL1β are considered pivotal factors in the pathogenesis of NAFLD. Several studies have demonstrated that TNF receptors (TNFR) deficiency, administration of anti-TNF or selective anti-TNFR1 antibody can ameliorate NAFLD in murine models [[33], [34], [35]]. Furthermore, Kamari et al. [36] reported that lack of IL1α or IL1β inhibits the progression of NASH in mice, while Mridha et al. [37] showed that the inhibitor of NLR family pyrin domain containing 3 (Nlrp3), which processes IL1β, reduces liver inflammation and fibrosis in experimental NASH in mice. However, the systemic administration of antibodies or small molecule drugs targeting TNF or IL1β often gives rise to side effects, such as severe infections, owing to their extensive tissue distribution. Previous studies have demonstrated that macrophages, as natural immune cells, play an important role in the progression of NASH by secreting pro-inflammatory factors such as TNF and IL1β [[5], [6], [7], [8]]. We conducted an analysis of liver mRNA profiles and single-cell RNA sequencing data from human NAFLD patients and mouse NASH models. The results revealed significantly elevated expression levels of TNF and IL1β in the livers of NASH patients or mice, with hepatic macrophages identified as the primary source (Fig. 1A–C).

Subsequently, we aimed to assess the viability of inhibiting TNF and IL1β expression in hepatic macrophages as a potential therapeutic approach for NASH. Several crucial considerations need to be carefully evaluated. Firstly, selecting appropriate drugs. To inhibit TNF, we selected ASO-TNF as a cost-effective alternative to TNF antibodies. In contrast to TNF SiRNA, ASO-TNF is a DNA molecule that exhibits enhanced stability. To inhibit IL1β, we chose a classical glycolysis inhibitor 2DG. Recent studies have revealed that pro-inflammatory macrophages primarily rely on glycolysis for energy metabolism and treatment with 2DG can effectively suppress macrophage glycolysis and significantly reduce IL1β expression in macrophages [[19], [20], [21]]. Additionally, our findings demonstrated that 2DG substantially inhibited TNF expression in macrophages (Fig. 1D–F). It is worth noting that chronic ingestion of 2DG resulted in cardiac vacuolization and increased mortality in rats [38], thereby emphasizing the imperative for targeted drug delivery. Secondly, the selection of an appropriate drug delivery system to target hepatic macrophages is crucial. Compared to exogenous nanocarriers, exosomes possess the advantages of higher biocompatibility and lower immunogenicity [11,12]. Importantly, both previous studies and our investigations have demonstrated that intravenously injected exosomes predominantly accumulate in the liver and are internalized by hepatic macrophages [10,[13], [14], [15]]. This suggests that exosomes may serve as a natural delivery platform for targeting hepatic macrophages. One of the current challenges is how to facilitate the escape of cargo delivered by exosomes from the endosomes into the cytoplasm, rather than being degraded in the lysosomes [39]. A focus of our future research involves investigating whether the introduction of pH-sensitive molecules, such as GALA peptides that form transmembrane peptide pores in acidic environments [40], can enhance endosomal escape of exosomes.

In this study, ASO-TNF was cholesterol-modified and subsequently incorporated into the exosomal membrane owing to its lipophilic properties, while small molecule drug 2DG was loaded into exosomes via electroporation. We further demonstrated that Exo/ASO-TNF or Exo/2DG effectively suppressed TNF and/or IL1β expression in macrophages *in vitro* and *in vivo*. Then we showed that infusion of Exo/ASO-TNF or Exo/2DG could alleviate experimental steatohepatitis in CDAA-fed or MCD-fed mice. We then asked whether exosomes loaded with both ASO-TNF and 2DG (Exo/ASO-TNF/2DG) exhibit enhanced therapeutic efficacy in NASH. But the results showed that infusion of Exo/ASO-TNF/2DG did not yield further improvements in NASH compared with Exo/ASO-TNF or Exo/2DG group in CDAA-fed mice (Fig. S8). This could potentially be attributed to the overlapping mechanisms between Exo/ASO-TNF and Exo/2DG, as both of which may work by inhibiting TNF and IL1β expression *in vivo*. Another point to consider is that Exo/ASO-CT is a more rigorous control for Exo/ASO-TNF. As shown in Fig. S9, the effects of Exo or Exo/ASO-CT infusion on CDAA-induced NASH were investigated, and the results revealed no significant difference between Exo and Exo/ASO-CT.

It is worth noting that Exo/ASO-TNF failed to reduce IL1β expression in macrophages *in vitro* (Fig. 3A–C), whereas treatment with Exo/ASO-TNF could decrease IL1β expression in livers or hepatic macrophages from NASH mice (Fig. 3, Fig. 5, Fig. 6). Does Exo/ASO-TNF influence the expression of IL1β by modulating Nlrp3 inflammasome activation *in vivo*? Western blot analysis was employed to detect the expression of proteins associated with Nlrp3 inflammasome activation. As shown in Fig. S10, no significant differences were observed in the expressions of Nlrp3, Caspase 1 (Casp1), Cleaved-Casp1 and Apoptosis associated speck like protein containing CARD (ASC) between the Exo/ASO-TNF group and the control group in CDAA-mice; however, a decrease was observed in the expressions of IL1β and Cleaved-IL1β. These results suggested that Exo/ASO-TNF may not down-regulate IL1β expression by affecting Nlrp3 inflammasome activation. Furthermore, GSEA analysis of RNA-seq data revealed that treatment with Exo/ASO-TNF inhibited TNF, Toll and MAPK inflammatory signaling pathways (Fig. 7A). Based on these results, we speculated that Exo/ASO-TNF may exert its effect on IL1β expression at the transcriptional level through inhibition of liver inflammation microenvironment.

Next, we explored the potential mechanisms underlying the inhibitory effects of Exo/ASO-TNF or Exo/2DG on NASH progression. Our findings revealed that these treatments effectively suppressed the expression of Pparγ and Acc1, key genes involved in fatty acid synthesis. It has been reported that hepatocyte-specific Pparγ knockout significantly inhibited NAFLD in mice [[41], [42], [43], [44]], which is consistent with our results. We performed RNA-seq analysis to compare hepatic mRNA profiles between Exo/ASO-TNF or Exo/2DG and Exo control in CDAA-fed model. Gene set enrichment analysis revealed that treatment with Exo/ASO-TNF or Exo/2DG resulted in down-regulation of inflammatory signaling pathways, including TNF, Toll, MAPK or IL17 signaling pathways (Fig. 7A). These findings suggested that Exo/ASO-TNF and Exo/2DG exhibited anti-inflammatory effects. On the other hand, Exo/ASO-TNF or Exo/2DG also induced the up-regulation of genes associated with oxidative phosphorylation and thermogenesis in the liver (Fig. 7A), thereby suggesting their potential to enhance fatty acid catabolism. Among the top 30 up-regulated genes observed in both the Exo/ASO-TNF and Exo/2DG treatment groups, we identified Sod1 as a downstream candidate gene based on its antioxidative and anti-inflammatory properties. Sod1 is one of the first line of antioxidant enzymes against oxidative stress [29], and previous studies have demonstrated that Sod1 knockout exacerbates NAFLD in mice [[26], [27], [28]]. Rescue experiments showed that inhibition of Sod1 counteracted the therapeutic effects of Exo/ASO-TNF or Exo/2DG on experimental NASH in mice (Fig. 8).

Researchers have also explored the use of alternative antioxidants for alleviating NAFLD. For instance, studies have found that the antioxidant N-acetylcysteine (NAC) can block hepatic lipid accumulation in preclinical models of NAFLD [[45], [46], [47]]. Luedde et al. [48] found that a diet supplemented with the antioxidant butylated hydroxyanisole (BHA) prevents the spontaneous development of steatohepatitis in NEMO^LPC−KO^ mice. Frank J. Gonzalez's research group found that Tempol, a SOD mimetic, can significantly attenuate NAFLD in mice [49]. Furthermore, our results also indicated that NAC, BHA, or Tempol could attenuate lipid accumulation in AML12 hepatocytes (Fig. S11). These antioxidants, however, still face several challenges that need to be addressed in order to facilitate their clinical translation. These include inadequate targeting and widespread distribution within the body. Exosomes exhibit excellent biocompatibility and liver-targeting properties, rendering them a promising drug delivery system for the treatment of liver diseases. This provides us with a research idea for our subsequent steps: using exosomes as carriers to encapsulate small molecule antioxidants, thereby selectively inhibiting oxidative stress in the liver and ultimately accomplishing the objective of treating acute or chronic liver injuries.

In summary, we demonstrated that exosomes loading with ASO-TNF or 2DG inhibited the expression of TNF or/and IL1β in macrophages *in vitro* and *in vivo*. Furthermore, infusion of Exo/ASO-TNF or Exo/2DG could reduce liver fat deposition, suppress liver inflammation, impede hepatic fibrosis, and ultimately alleviate experimental steatohepatitis in CDAA-fed or MCD-fed mice. Mechanistically, we found that treatment of Exo/ASO-TNF or Exo/2DG potentially alleviated the progression of experimental NASH by up-regulating the expression of Sod1 (Fig. S12). Thus, infusion of exosomes loaded with ASO-TNF or 2DG represents a promising therapeutic strategy for NASH treatment that merits further investigation.

## CRediT authorship contribution statement

**Fei He:** Writing – original draft, Investigation, Funding acquisition, Conceptualization. **Wei Du:** Methodology, Investigation. **Yingying Liu:** Methodology, Investigation. **Yuwei Ling:** Visualization, Software. **Ming Xu:** Methodology, Investigation. **Jingjing Liu:** Methodology. **Ping Song:** Methodology, Funding acquisition. **Zhiqiang Fang:** Methodology. **Zhensheng Yue:** Methodology, Data curation. **Juanli Duan:** Methodology, Formal analysis. **Lin Wang:** Writing – review & editing, Supervision, Project administration, Funding acquisition, Conceptualization.

## Ethical approval

All animal procedures used in this study were approved by the Ethics Committee of Fourth Military Medical University.

## Availability of data and materials

All data are available in the main text and are available from the corresponding authors upon reasonable request.

## Funding

This work was supported by grants from 10.13039/501100001809National Natural Science Foundation of China (82325007; 92468202; 81970535; 82300703), the 10.13039/501100012166National Key Research and Development Program of China (2021YFA1100502), and the Xijing Hospital Project (LHJJ24JH29; XJZT24QN10).

## Declaration of competing interest

The authors declare that they have no known competing financial interests or personal relationships that could have appeared to influence the work reported in this paper.

## Data Availability

Data will be made available on request.

## References

[1] Younossi Z.M., Koenig A.B., Abdelatif D., Fazel Y., Henry L., Wymer M. (2016). Global epidemiology of nonalcoholic fatty liver disease-Meta-analytic assessment of prevalence, incidence, and outcomes. Hepatology.

[2] Diehl A.M., Day C. (2017). Cause, pathogenesis, and treatment of nonalcoholic steatohepatitis. N. Engl. J. Med..

[3] Powell E.E., Wong V.W., Rinella M. (2021). Non-alcoholic fatty liver disease. Lancet..

[4] Heymann F., Tacke F. (2016). Immunology in the liver--from homeostasis to disease. Nat. Rev. Gastroenterol. Hepatol..

[5] Miura K., Kodama Y., Inokuchi S., Schnabl B., Aoyama T., Ohnishi H. (2010). Toll-like receptor 9 promotes steatohepatitis by induction of interleukin-1beta in mice. Gastroenterology.

[6] Stienstra R., Saudale F., Duval C., Keshtkar S., Groener J.E., van Rooijen N. (2010). Kupffer cells promote hepatic steatosis via interleukin-1beta-dependent suppression of peroxisome proliferator-activated receptor alpha activity. Hepatology.

[7] Diehl K.L., Vorac J., Hofmann K., Meiser P., Unterweger I., Kuerschner L. (2020). Kupffer cells sense free fatty acids and regulate hepatic lipid metabolism in high-fat diet and inflammation. Cells.

[8] Tosello-Trampont A.C., Landes S.G., Nguyen V., Novobrantseva T.I., Hahn Y.S. (2012). Kuppfer cells trigger nonalcoholic steatohepatitis development in diet-induced mouse model through tumor necrosis factor-α production. J. Biol. Chem..

[9] Zhang X., Fan L., Wu J., Xu H., Leung W.Y., Fu K. (2019). Macrophage p38α promotes nutritional steatohepatitis through M1 polarization. J. Hepatol..

[10] Ding J., Xu M., Du W., Fang Z.Q., Xu H., Liu J.J. (2023). Myeloid-specific blockade of Notch signaling ameliorates nonalcoholic fatty liver disease in mice. Int. J. Biol. Sci..

[11] Valadi H., Ekström K., Bossios A., Sjöstrand M., Lee J.J., Lötvall J.O. (2007). Exosome-mediated transfer of mRNAs and microRNAs is a novel mechanism of genetic exchange between cells. Nat. Cell Biol..

[12] EL Andaloussi S., Mäger I., Breakefield X.O., Wood M.J. (2013). Extracellular vesicles: biology and emerging therapeutic opportunities. Nat. Rev. Drug Discov..

[13] Imai T., Takahashi Y., Nishikawa M., Kato K., Morishita M., Yamashita T. (2015). Macrophage-dependent clearance of systemically administered B16BL6-derived exosomes from the blood circulation in mice. J. Extracell. Vesicles.

[14] Zhang G., Huang X., Xiu H., Sun Y., Chen J., Cheng G. (2020). Extracellular vesicles: natural liver-accumulating drug delivery vehicles for the treatment of liver diseases. J. Extracell. Vesicles.

[15] He F., Li W.N., Li X.X., Yue K.Y., Duan J.L., Ruan B. (2022). Exosome-mediated delivery of RBP-J decoy oligodeoxynucleotides ameliorates hepatic fibrosis in mice. Theranostics.

[16] Myers K.J., Murthy S., Flanigan A., Witchell D.R., Butler M., Murray S. (2003). Antisense oligonucleotide blockade of tumor necrosis factor-alpha in two murine models of colitis. J. Pharmacol. Exp. Therapeut..

[17] Zuo L., Huang Z., Dong L., Xu L., Zhu Y., Zeng K. (2010). Targeting delivery of anti-TNFalpha oligonucleotide into activated colonic macrophages protects against experimental colitis. Gut.

[18] Huang Z., Zhang Z., Zha Y., Liu J., Jiang Y., Yang Y. (2012). The effect of targeted delivery of anti-TNF-α oligonucleotide into CD169+ macrophages on disease progression in lupus-prone MRL/lpr mice. Biomaterials.

[19] Rodríguez-Prados J.C., Través P.G., Cuenca J., Rico D., Aragonés J., Martín-Sanz P. (2010). Substrate fate in activated macrophages: a comparison between innate, classic, and alternative activation. J. Immunol..

[20] Tannahill G.M., Curtis A.M., Adamik J., Palsson-McDermott E.M., McGettrick A.F., Goel G. (2013). Succinate is an inflammatory signal that induces IL-1β through HIF-1α. Nature.

[21] Freemerman A.J., Johnson A.R., Sacks G.N., Milner J.J., Kirk E.L., Troester M.A. (2014). Metabolic reprogramming of macrophages: glucose transporter 1 (GLUT1)-mediated glucose metabolism drives a proinflammatory phenotype. J. Biol. Chem..

[22] Didiot M.C., Hall L.M., Coles A.H., Haraszti R.A., Godinho B.M., Chase K. (2016). Exosome-mediated delivery of hydrophobically modified siRNA for huntingtin mRNA silencing. Mol. Ther..

[23] Yerneni S.S., Lathwal S., Shrestha P., Shirwan H., Matyjaszewski K., Weiss L. (2019). Rapid on-demand extracellular vesicle augmentation with versatile oligonucleotide tethers. ACS Nano.

[24] Gong C., Tian J., Wang Z., Gao Y., Wu X., Ding X. (2019). Functional exosome-mediated co-delivery of doxorubicin and hydrophobically modified microRNA 159 for triple-negative breast cancer therapy. J. Nanobiotechnol..

[25] Takada D., Emi M., Ezura Y., Nobe Y., Kawamura K., Iino Y. (2002). Interaction between the LDL-receptor gene bearing a novel mutation and a variant in the apolipoprotein A-II promoter: molecular study in a 1135-member familial hypercholesterolemia kindred. J. Hum. Genet..

[26] Uchiyama S., Shimizu T., Shirasawa T. (2006). CuZn-SOD deficiency causes ApoB degradation and induces hepatic lipid accumulation by impaired lipoprotein secretion in mice. J. Biol. Chem..

[27] Wang L., Jiang Z., Lei X.G. (2012). Knockout of SOD1 alters murine hepatic glycolysis, gluconeogenesis, and lipogenesis. Free Radic. Biol. Med..

[28] Sakiyama H., Fujiwara N., Yoneoka Y., Yoshihara D., Eguchi H., Suzuki K. (2016). Cu,Zn-SOD deficiency induces the accumulation of hepatic collagen. Free Radic. Res..

[29] Jomova K., Alomar S.Y., Alwasel S.H., Nepovimova E., Kuca K., Valko M. (2024). Several lines of antioxidant defense against oxidative stress: antioxidant enzymes, nanomaterials with multiple enzyme-mimicking activities, and low-molecular-weight antioxidants. Arch. Toxicol..

[30] Afonso V., Santos G., Collin P., Khatib A.M., Mitrovic D.R., Lomri N. (2006). Tumor necrosis factor-alpha down-regulates human Cu/Zn superoxide dismutase 1 promoter via JNK/AP-1 signaling pathway. Free Radic. Biol. Med..

[31] Abreu I.A., Cabelli D.E. (2010). Superoxide dismutases-a review of the metal-associated mechanistic variations. Biochim. Biophys. Acta.

[32] Arroyave-Ospina J.C., Wu Z., Geng Y., Moshage H. (2021). Role of oxidative stress in the pathogenesis of non-alcoholic fatty liver disease: implications for prevention and therapy. Antioxidants.

[33] Tomita K., Tamiya G., Ando S., Ohsumi K., Chiyo T., Mizutani A. (2006). Tumour necrosis factor alpha signalling through activation of Kupffer cells plays an essential role in liver fibrosis of non-alcoholic steatohepatitis in mice. Gut.

[34] Li Z., Yang S., Lin H., Huang J., Watkins P.A., Moser A.B. (2003). Probiotics and antibodies to TNF inhibit inflammatory activity and improve nonalcoholic fatty liver disease. Hepatology.

[35] Wandrer F., Liebig S., Marhenke S., Vogel A., John K., Manns M.P. (2020). TNF-Receptor-1 inhibition reduces liver steatosis, hepatocellular injury and fibrosis in NAFLD mice. Cell Death Dis..

[36] Kamari Y., Shaish A., Vax E., Shemesh S., Kandel-Kfir M., Arbel Y. (2011). Lack of interleukin-1α or interleukin-1β inhibits transformation of steatosis to steatohepatitis and liver fibrosis in hypercholesterolemic mice. J. Hepatol..

[37] Mridha A.R., Wree A., Robertson A.A.B., Yeh M.M., Johnson C.D., Van Rooyen D.M. (2017). NLRP3 inflammasome blockade reduces liver inflammation and fibrosis in experimental NASH in mice. J. Hepatol..

[38] Minor R.K., Smith D.L., Sossong A.M., Kaushik S., Poosala S., Spangler E.L. (2010). Chronic ingestion of 2-deoxy-D-glucose induces cardiac vacuolization and increases mortality in rats. Toxicol. Appl. Pharmacol..

[39] Amina S.J., Azam T., Dagher F., Guo B. (2024). A review on the use of extracellular vesicles for the delivery of drugs and biological therapeutics. Expet Opin. Drug Deliv..

[40] Li W., Nicol F., Szoka F.C. (2004). GALA: a designed synthetic pH-responsive amphipathic peptide with applications in drug and gene delivery. Adv. Drug Deliv. Rev..

[41] Morán-Salvador E., López-Parra M., García-Alonso V., Titos E., Martínez-Clemente M., González-Périz A. (2011). Role for PPARγ in obesity-induced hepatic steatosis as determined by hepatocyte- and macrophage-specific conditional knockouts. Faseb. J..

[42] Cordoba-Chacon J. (2020). Loss of hepatocyte-specific PPARγ expression ameliorates early events of steatohepatitis in mice fed the methionine and choline-deficient diet. PPAR Res..

[43] Lee S.M., Pusec C.M., Norris G.H., De Jesus A., Diaz-Ruiz A., Muratalla J. (2021). Hepatocyte-specific loss of PPARγ protects mice from NASH and increases the therapeutic effects of rosiglitazone in the liver. Cell Mol Gastroenterol Hepatol.

[44] Gavrilova O., Haluzik M., Matsusue K., Cutson J.J., Johnson L., Dietz K.R. (2003). Liver peroxisome proliferator-activated receptor gamma contributes to hepatic steatosis, triglyceride clearance, and regulation of body fat mass. J. Biol. Chem..

[45] Dludla P.V., Nkambule B.B., Mazibuko-Mbeje S.E., Nyambuya T.M., Marcheggiani F., Cirilli I. (2020). N-acetyl cysteine targets hepatic lipid accumulation to curb oxidative stress and inflammation in NAFLD: a comprehensive analysis of the literature. Antioxidants.

[46] Lin C.C., Yin M.C., Hsu C.C., Lin M.P. (2004). Effect of five cysteine-containing compounds on three lipogenic enzymes in Balb/cA mice consuming a high saturated fat diet. Lipids.

[47] Samuhasaneeto S., Thong-Ngam D., Kulaputana O., Patumraj S., Klaikeaw N. (2007). Effects of N-acetylcysteine on oxidative stress in rats with non-alcoholic steatohepatitis. J. Med. Assoc. Thai..

[48] Luedde T., Beraza N., Kotsikoris V., van Loo G., Nenci A., De Vos R. (2007). Deletion of NEMO/IKKgamma in liver parenchymal cells causes steatohepatitis and hepatocellular carcinoma. Cancer Cell.

[49] Jiang C., Xie C., Li F., Zhang L., Nichols R.G., Krausz K.W. (2015). Intestinal farnesoid X receptor signaling promotes nonalcoholic fatty liver disease. J. Clin. Invest..

