# Supplementary material for: Exosome-equipped TNF antisense oligodeoxynucleotide or 2-deoxy-D-glucose ameliorated nonalcoholic steatohepatitis by modulating superoxide dismutase 1 in mice
Source: Redox Biol. 2025 Jan 3;80:103488. doi: 10.1016/j.redox.2025.103488 (PMC11763583; doi:10.1016/j.redox.2025.103488)
Supplement: Multimedia component 1 [file mmc1.docx]

**Exosome-equipped** **TNF antisense oligodeoxynucleotide or** **2-deoxy-D-glucose** **ameliorated** **nonalcoholic steatohepatitis by modulating superoxide dismutase 1 in mice**

Fei He**^*, #^** (hefei_hefei@163.com)

Wei Du**^*^** (dw41@qq.com)

Yingying Liu**^*^** (15231860278@163.com)

Yuwei Ling (henryling602@163.com)

Ming Xu (1693538788@qq.com)

Jingjing Liu (ljj05080086@126.com)

Ping Song ([songping000123@163.com](mailto:songping000123@163.com))

Zhiqiang Fang ([zhiqiang_fang@163.com](mailto:zhiqiang_fang@163.com))

Zhensheng Yue (18392181767@163.com)

Juanli Duan**^#^** ([duan_juan_li@126.com](mailto:duan_juan_li@126.com))

Lin Wang**^#^** ([fierywang@163.com](mailto:fierywang@163.com))

Department of Hepatobiliary Surgery, Xijing Hospital, Fourth Military Medical University, Xi’an 710032, China.

***** These authors contributed equally to this study.

**^#^** Correspondance to**:** Lin Wang ([fierywang@163.com](mailto:fierywang@163.com)), Juanli Duan ([duan_juan_li@126.com](mailto:duan_juan_li@126.com)) and Fei He (hefei_hefei@163.com)

**Supplementary materials and methods**

***Detection of Reactive Oxygen Species (ROS), mitochondrial superoxides or superoxide anion in hepatocytes***

The liver single-cell suspension from MCD-fed mice was prepared by perfusion with collagenase IV (0.2 g L^-1^; Sigma). Subsequently, hepatocytes were harvested through centrifugation for 3 min at a speed of 50 × g. The level of ROS in primary mouse hepatocytes were detected using Reactive Oxygen Species Assay Kit (Beyotime Biotechnology). Briefly, primary mouse hepatocytes (at a final cell density of 5 × 10^6^ cells mL^-1^) were incubated with DCFH-DA (10 μM) at 37℃ for 20 min. The cell resuspension was mixed every 5 minutes to ensure adequate contact between probes and cells, and then the cells were washed three times to thoroughly remove any residual DCFH-DA. Subsequently, the levels of ROS were quantified using flow cytometry.

The level of mitochondrial superoxides in AML12 cells were measured using Mitochondrial Superoxide Assay Kit with MitoSOX Red (Beyotime Biotechnology). Briefly, dissociated AML12 (at a final cell density of 5 × 10^6^ cells mL^-1^) were incubated with MitoSOX Red (5 μM) at 37℃ for 20 min. The cells were then washed three times to remove any residual MitoSOX Red and the levels of mitochondrial superoxides were quantified using flow cytometry. For adherent AML12 cells, the culture medium was removed and the cells were washed with PBS before being incubated with PBS containing MitoSOX Red (5 μM) at 37℃ for 20 min. After incubation, the supernatant was removed and the cells were washed twice with PBS. Photomicrographs were captured using a fluorescence microscope (BX51, Olympus).

The level of superoxide anion in AML12 cells were measured using Superoxide Assay Kit (Beyotime Biotechnology). Briefly, AML12 cells (1 × 10^4^ cells per well in a 96-well plate) were cultured with conditioned medium from RAW264.7 cells, either with or without LCS-1 (2 µM), in palmitic acid medium (PA, 200 µM) for 24 hours. The culture medium was aspirated and the cells were washed with PBS. Then, 200 μl of superoxide assay working solution was added to each well and incubated at 37℃ for 1 hour. The absorbance at 450 nm was measured.

***Detection of adenosine 5'-triphosphate (ATP) content in AML12 cells***

The ATP level in AML12 cells was quantified using an ATP assay kit (Nanjing Jiancheng Bioengineering Institute, Nanjing, China). AML12 cells (1 × 10^6^ cells) were harvested and suspended in 300-500 μl of cold water before being homogenized in an ice water bath (a portion was taken out to determine the protein concentration). Subsequently, the cell suspension was subjected to heat treatment in a boiling water bath for 10 minutes. After vortexing for 1 minute, the resulting suspension was utilized for determination following the manufacturer's instructions.

***Detection of hydrogen peroxide (******H_2_O_2_) content in liver***

The content of H_2_O_2_ in liver samples was determined using Hydrogen Peroxide Assay Kit (Beyotime Biotechnology) following the manufacturer's instructions. Briefly, 200 μl of Lysis Buffer per 10 mg of liver tissue was added and homogenized. The mixture was then centrifuged at 12000 × g at 4℃ for 5 min. Subsequently, 50 μl of sample and 100 μl of H_2_O_2_ assay buffer were added to each well and incubated at room temperature for 30 minutes. The absorbance at 560 nm was measured to calculate the content of H_2_O_2_ based on the standard curve. Furthermore, the protein concentration in the liver was quantified using the BCA Protein Assay Kit (Beyotime) following the manufacturer’s instructions. Finally, the content of H_2_O_2_ in the liver per unit weight was calculated.

**Supplementary figures**

**
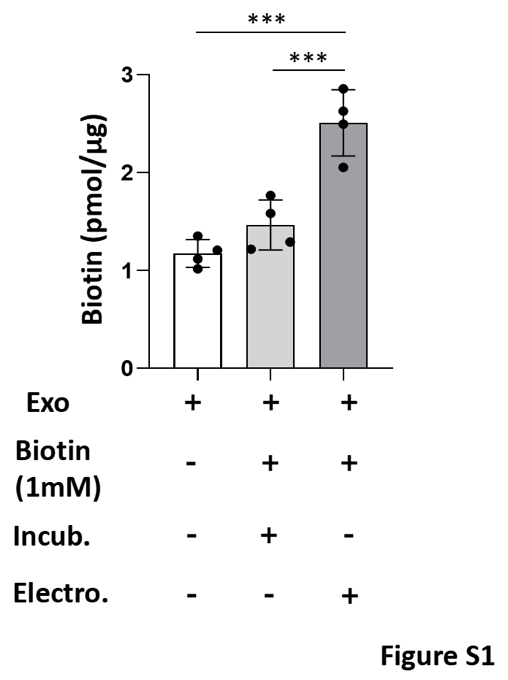
**

**Figure S1**

**Biotin was loaded into exosomes by electroporation.**

Biotin was loaded into exosomes (1 mM biotin *VS.* 50 µg exosomes) by electroporation or incubation, and the content of biotin in exosomes was detected using FITC labeled Streptavidin. Bars = means ± SD, ** P < 0.01, *** P < 0.001.

**
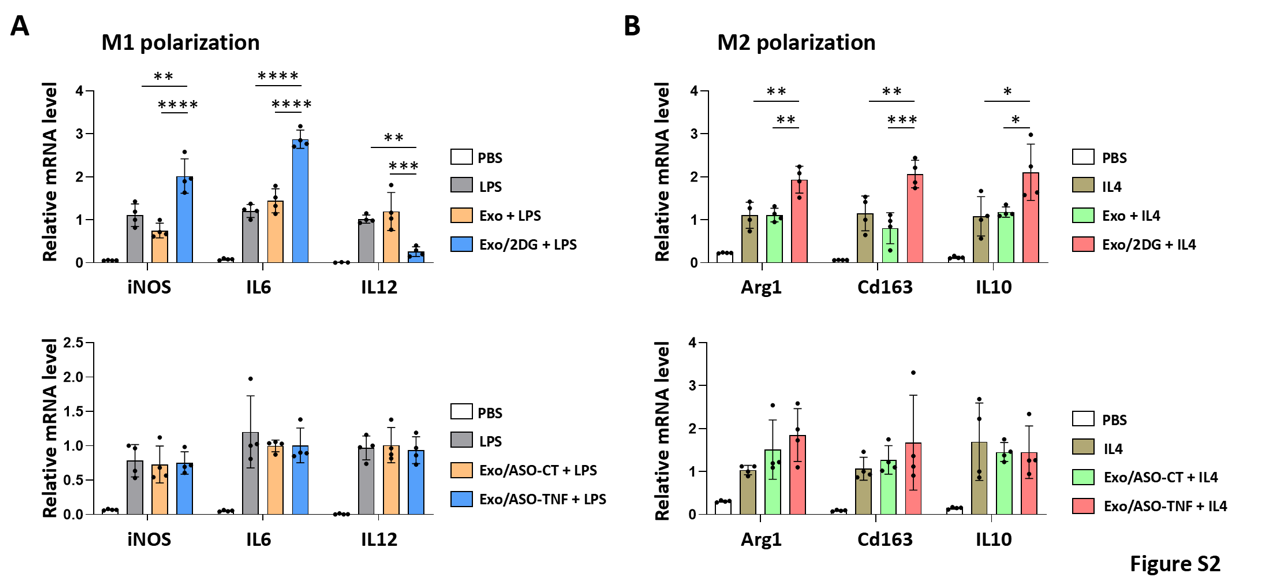
**

**Figure S2**

**Effects of Exo/ASO-TNF or Exo/2DG on the polarization of macrophages *in vitro*.** (A) BMDMs were incubated with Exo, Exo/2DG, Exo/ASO-CT or Exo/ASO-TNF for 24 h, then changed and cultured with the fresh medium containing LPS (200 ng mL^-1^) for another 24 h, and the expression of iNOS, IL6 and IL12 was assessed using qRT-PCR.

(B) BMDMs were treated with Exo, Exo/2DG, Exo/ASO-CT or Exo/ASO-TNF for 24 h, then stimulated with IL4 (10 ng mL^-1^) for another 24 h, and the expression of Arg1, Cd163 and IL10 was detected by qRT-PCR. Bars = means ± SD, * P < 0.05, ** P < 0.01, *** P < 0.001, **** P < 0.0001.

**
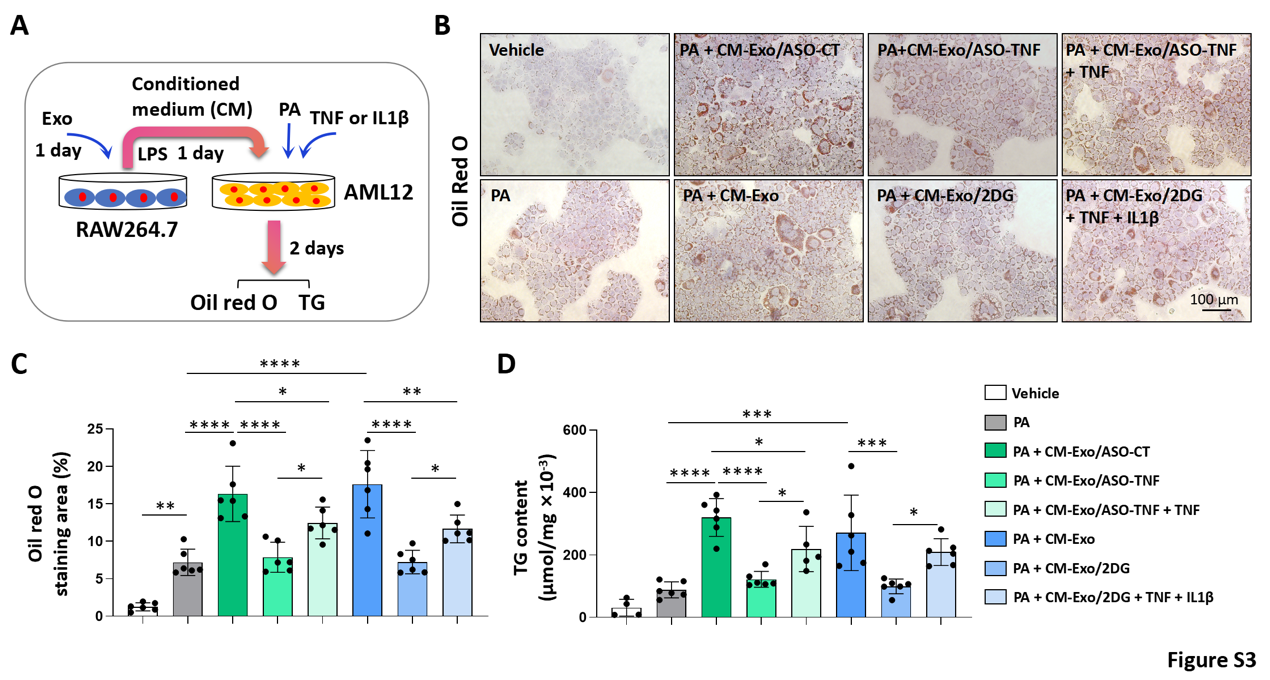
**

**Figure S3**

**Exo/ASO-TNF or Exo/2DG** **attenuated lipid accumulation in AML12 hepatocytes *in vitro*.**

(A) Schematic showing that RAW264.7 macrophages were treated with Exo/ASO-TNF or Exo/2DG for 1 day, changed and cultured with the fresh medium containing LPS for 1 day, and the conditioned medium (CM) were harvested. Then AML12 hepatocytes were cultured with CM, in the presence or absence of TNF (10 ng mL^-1^), and/or IL1β (10 ng mL^-1^) in palmitic acid medium (PA, 200 µM) for 2 days.

(B) Lipid accumulation in AML12 cells was assessed using oil red O staining.

(C) The positive areas of oil red O staining in (B) were quantitatively compared.

(D) Triglyceride (TG) content in AML12 hepatocytes was measured. Bars = means ± SD, * P < 0.05, ** P < 0.01, *** P < 0.001, **** P < 0.0001.

**
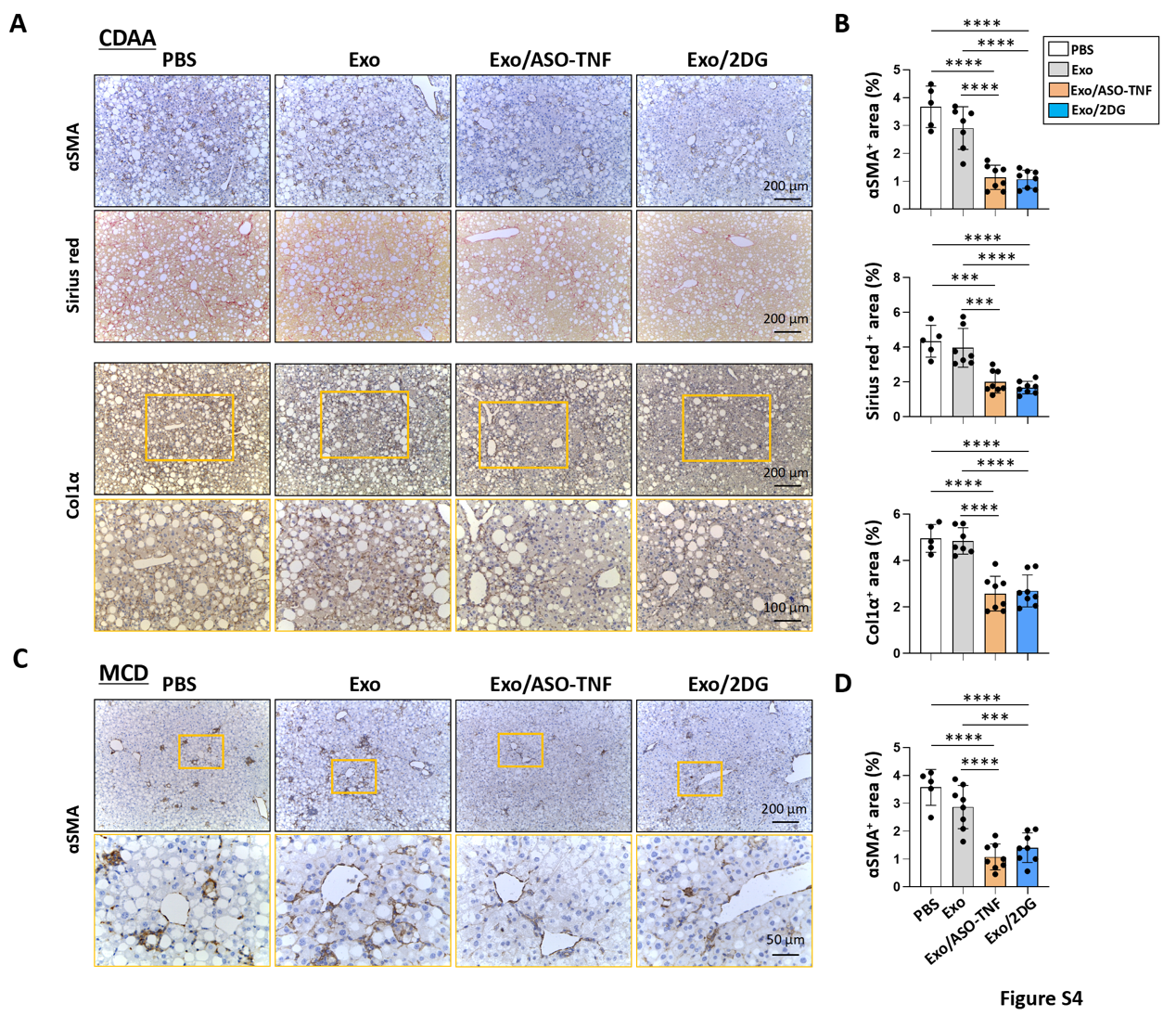
**

**Figure S4**

**Infusion of Exo/ASO-TNF or Exo/2DG attenuated experimental steatohepatitis-induced hepatic fibrosis in CDAA or MCD-fed mice.**

(A) Liver sections of CDAA-fed mice were stained with Sirius Red, or immunohistochemistry with anti-αSMA or anti-Col1α. (B) Positive signals for Sirius Red or immunohistochemistry staining were quantitatively compared. (C,D) Liver sections of MCD-fed mice were stained with anti-αSMA, and the positive areas of αSMA staining were quantitatively compared. Bars = means ± SD, *** P < 0.001, **** P < 0.0001.

**
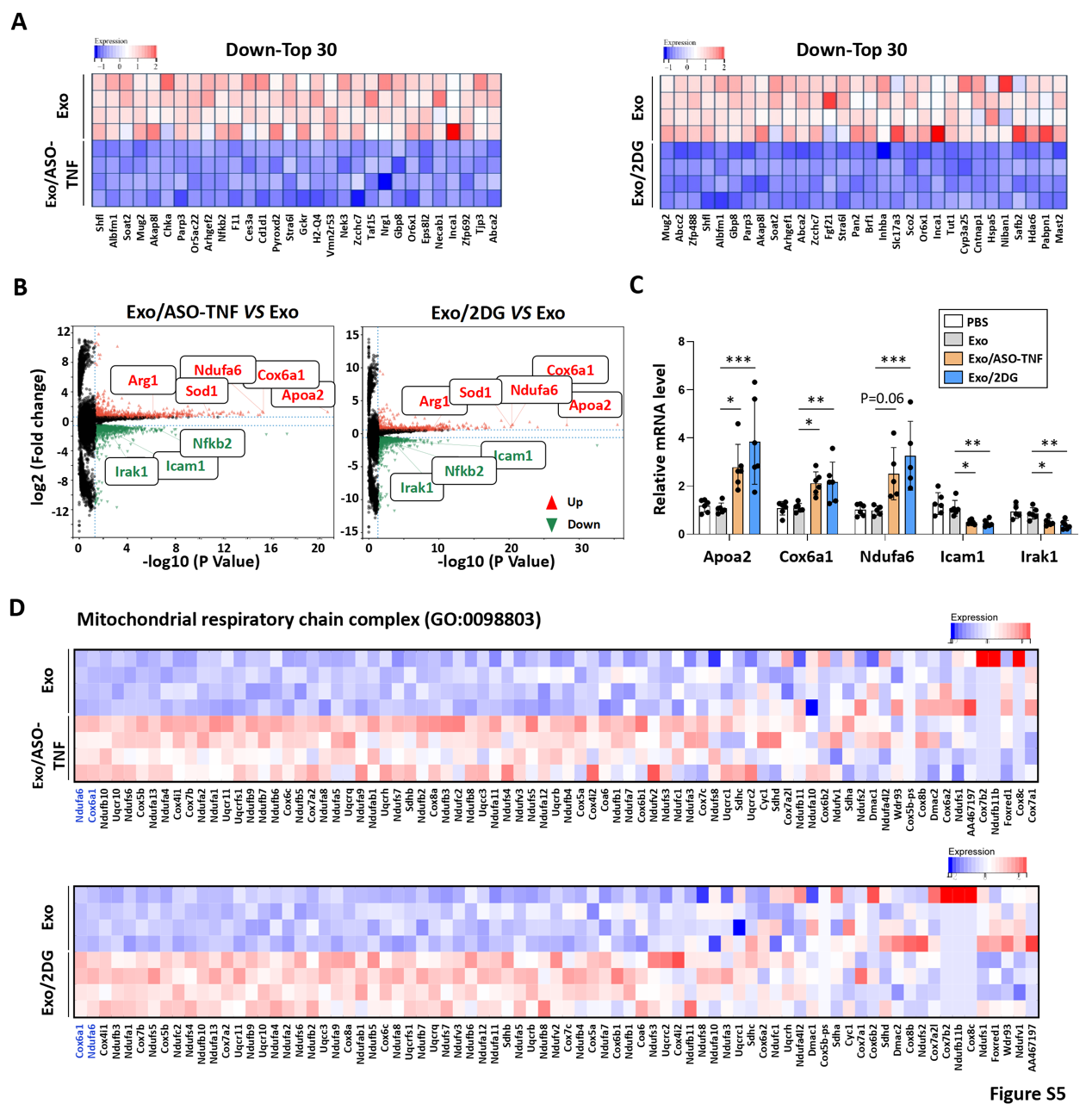
**

**Figure S5**

**The mRNA expression of liver samples from Exo/ASO-TNF, Exo/2DG or Exo-treated CDAA-fed mice was profiled by using RNA-seq.**

(A) Heatmaps showed top 30 down-regulated genes in the RNA-seq data between Exo/ASO-TNF, or Exo/2DG and Exo groups.

(B) The volcano plot showed the differentially expressed genes between Exo/ASO-TNF, or Exo/2DG and Exo groups.

(C) Expression of Apoa2, Cox6a1, Ndufa6, Icam1, and Irak1 was determined by qRT-PCR. Bars = means ± SD; * P < 0.05, ** P < 0.01, *** P < 0.001.

(D) Heatmaps showed mitochondrial respiratory chain complex associated genes (GO:0098803) in the RNA-seq data between Exo/ASO-TNF, or Exo/2DG and Exo groups.

**
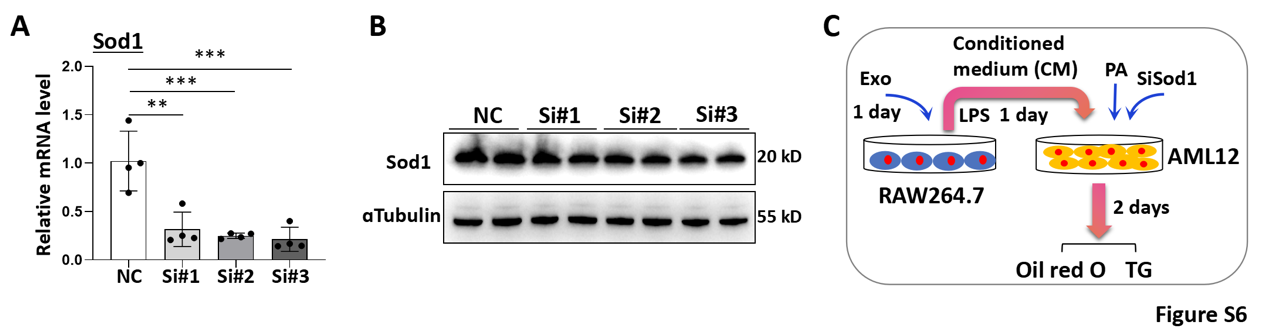
**

**Figure S6**

**Detection of Sod1 knockdown in AML12 and schematic illustration of rescue experiment *in vitro.***

(A,B) AML12 cells were transfected with SiSod1 (Si#1-3, 50 nM) or control SiRNA (NC) with lipofectamine 2000. And 48 h later, the mRNA level of Sod1 was detected by qRT-PCR (A), and the protein level of Sod1 was determined using Western blot.

(C) Schematic showing that the conditioned medium (CM) of RAW264.7 macrophages were harvested according to the above steps, then AML 12 hepatocytes were transfected with Sod1 siRNA, and 12 h later, cultured with CM and fresh palmitic acid medium for 2 days. Bars = means ± SD; ** P < 0.01, *** P < 0.001.

**
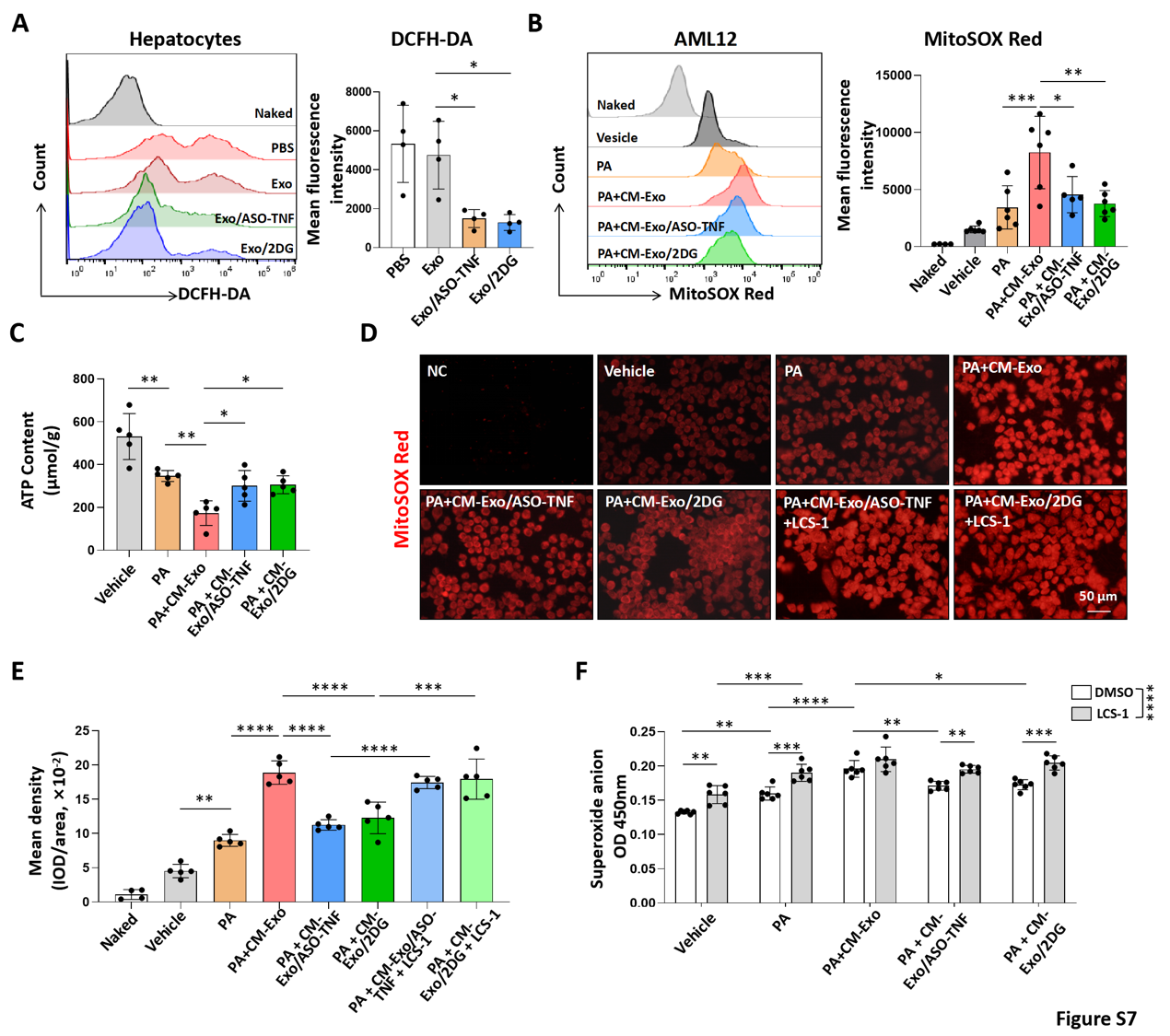
**

**Figure S7**

**Exo/ASO-TNF or Exo/2DG decreased the level of reactive oxygen species (ROS) in hepatocytes**

(A) Primary hepatocytes were isolated from the livers of PBS, Exo, Exo/ASO-TNF or Exo/2DG-treated MCD-fed mice. Subsequently, the isolated hepatocytes were incubated with DCFH-DA (10 µM) for 20 min, and the levels of ROS were determined using flow cytometry. The mean fluorescence intensity were quantitatively compared.

(B) RAW264.7 macrophages were treated with Exo/ASO-TNF or Exo/2DG for 24 hours, changed and cultured with the fresh medium containing LPS for another 24 hours, and the conditioned medium (CM) were collected. AML12 hepatocytes were then cultured with CM in palmitic acid medium (PA, 200 µM) for 48 hours. The treated AML12 hepatocytes were incubated with MitoSOX Red (5 µM) for 20 min, and the levels of superoxides in mitochondria were assessed using flow cytometry. The Mean fluorescence intensity were quantitatively compared.

(C) Detection of ATP content in AML12 cells.

(D-E) AML12 cells were stained with MitoSOX Red. Representative images were obtained by fluorescence microscopy (D). The Mean fluorescence intensity were quantitatively compared (E).

(F) The levels of superoxide anion in the treated AML12 hepatocytes were quantified by measuring absorbance at 450 nm. Analyzed by two-way ANOVA with Tukey’s post hoc analysis. Bars = means ± SD, * P < 0.05, ** P < 0.01, *** P < 0.001, **** P < 0.0001.

**
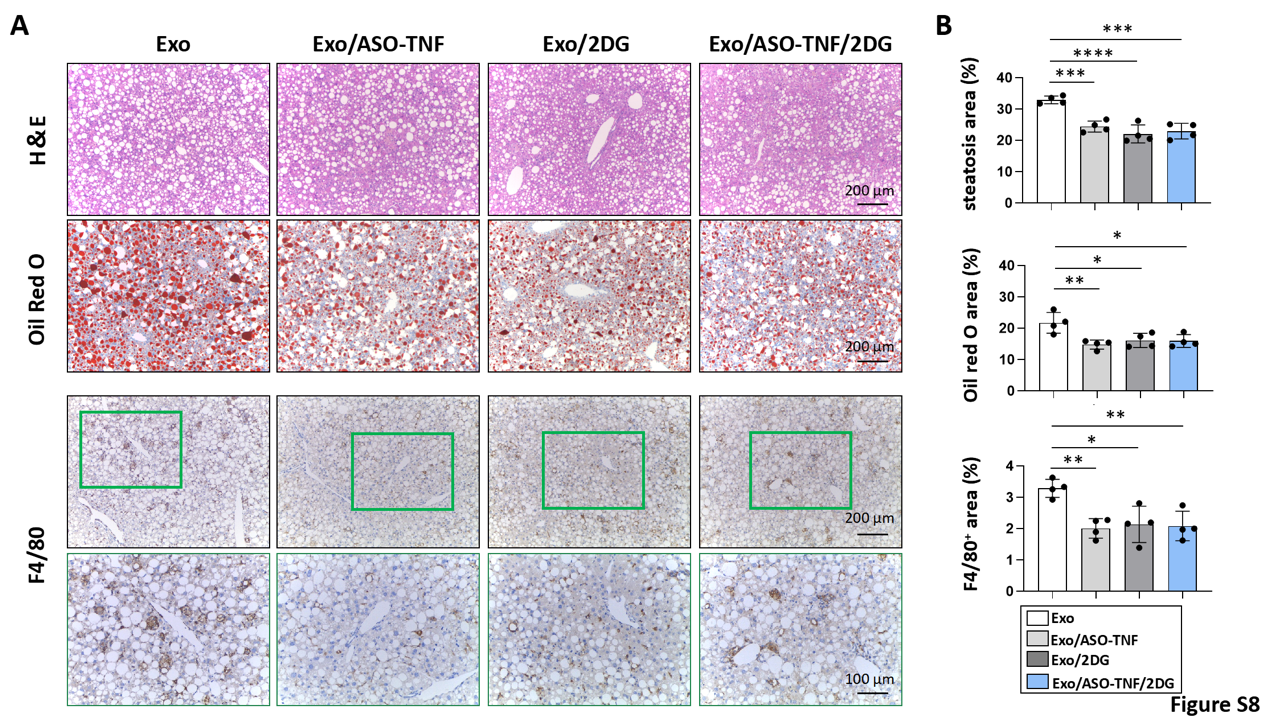
**

**Figure S8**

**The therapeutic efficacy of infusion exosomes co-loaded with 2DG and ASO-TNF did not exhibit a significant enhancement in experimental steatohepatitis in CDAA-fed mice compared to Exo/2DG or Exo/ASO-TNF.**

Mice were fed with CDAA diet for 10 weeks, and Exo, Exo/ASO-TNF, Exo/2DG or Exo/ASO-TNF/2DG (200 µg) were injected into mice four times via tail vein at the last 2 weeks. (A) Liver sections were subjected to H&E, Oil Red O, or immunohistochemical staining for F4/80. (B) Steatosis areas, positive signals for Oil Red O or immunohistochemistry staining were quantitatively compared. Bars = means ± SD, * P < 0.05, ** P < 0.01, *** P < 0.001, **** P < 0.0001.

**
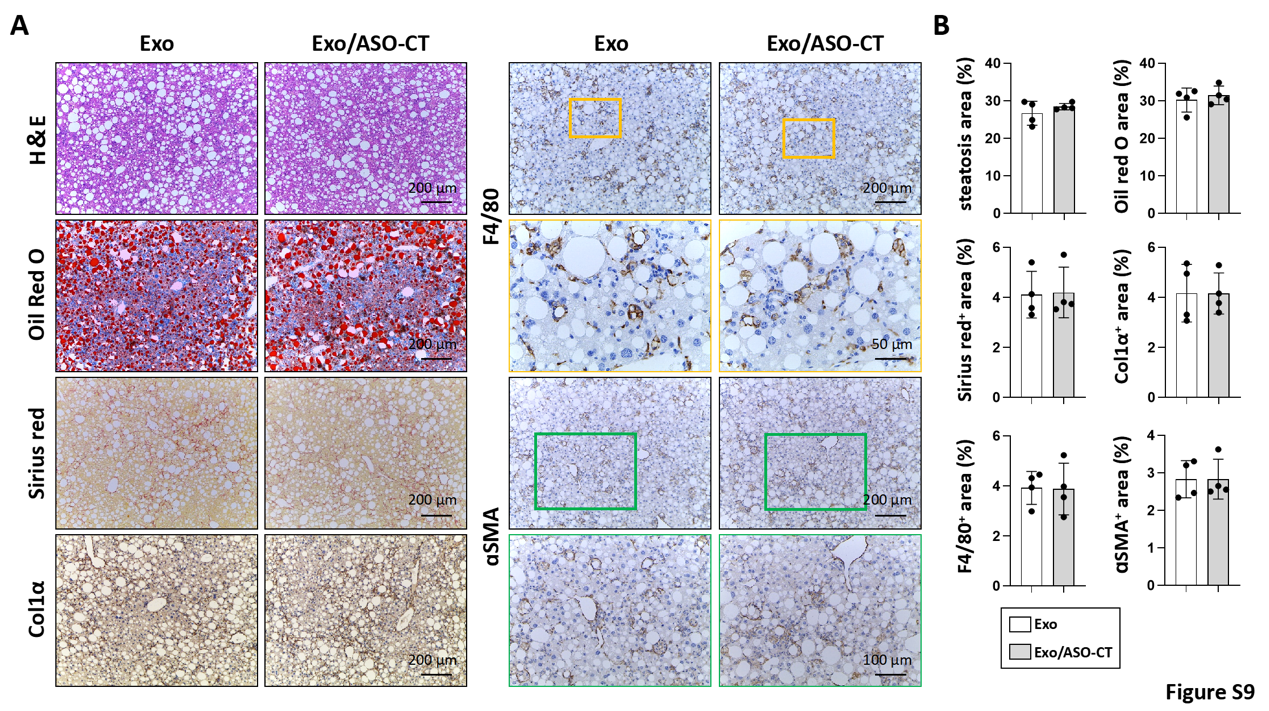
**

**Figure S9**

**No significant difference observed in the experimental steatohepatitis of CDAA-fed mice between Exo and Exo/ASO-CT treatment.**

Mice were fed with CDAA diet for 10 weeks, and Exo or Exo/ASO-CT (200 µg) were injected into mice four times via tail vein at the last 2 weeks. (A) Liver sections were subjected to H&E, Oil Red O, Sirius Red staining, or immunohistochemical staining for Col1α, F4/80, αSMA. (B) Steatosis areas, positive signals for Oil Red O, Sirius Red or immunohistochemistry staining were quantitatively compared. Bars = means ± SD.


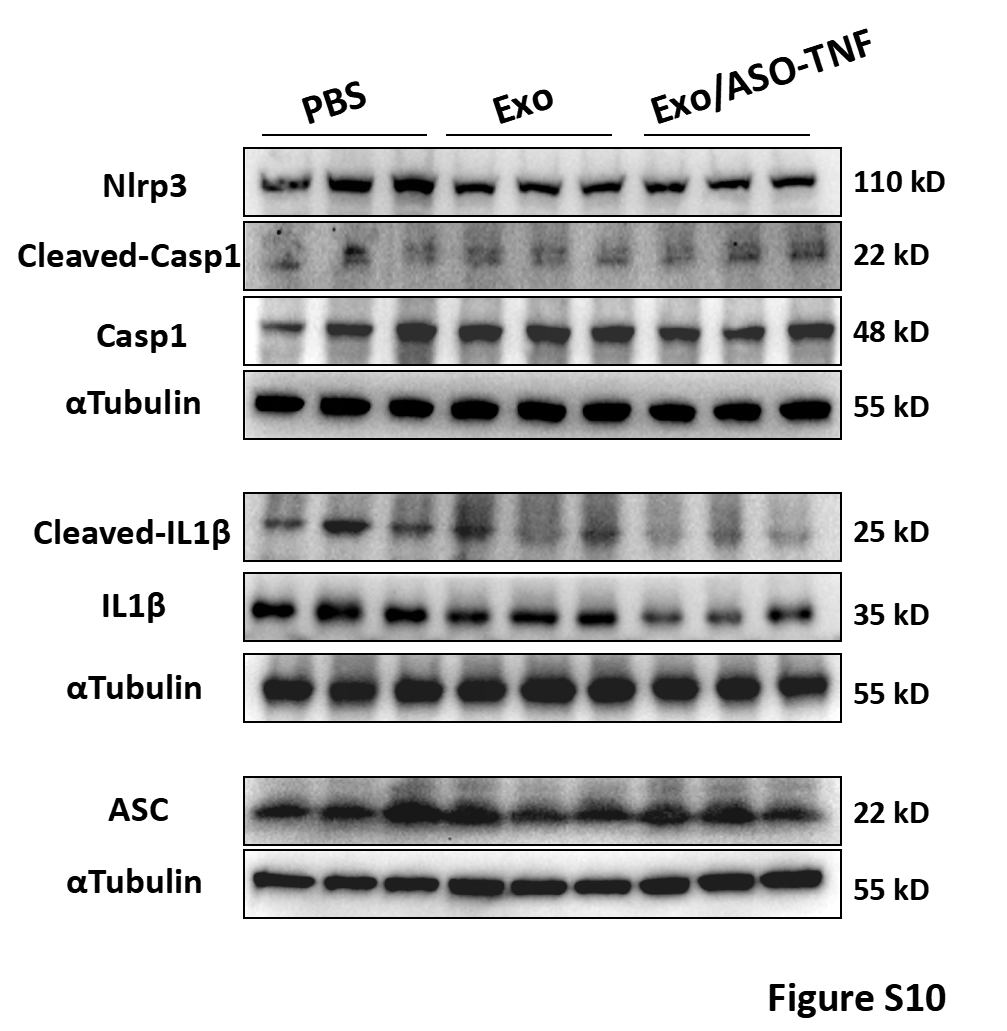


**Figure S10**

**Effects of Exo/ASO-TNF infusion on the activation of hepatic** **Nlrp3 inflammasome in CDAA-fed mice.**

The protein levels of Nlrp3, Caspase 1, Cleaved-Caspase 1, IL1β, Cleaved-IL1β, ASC and ɑTubulin in livers of CDAA-fed mice were assessed using Western blot analysis.


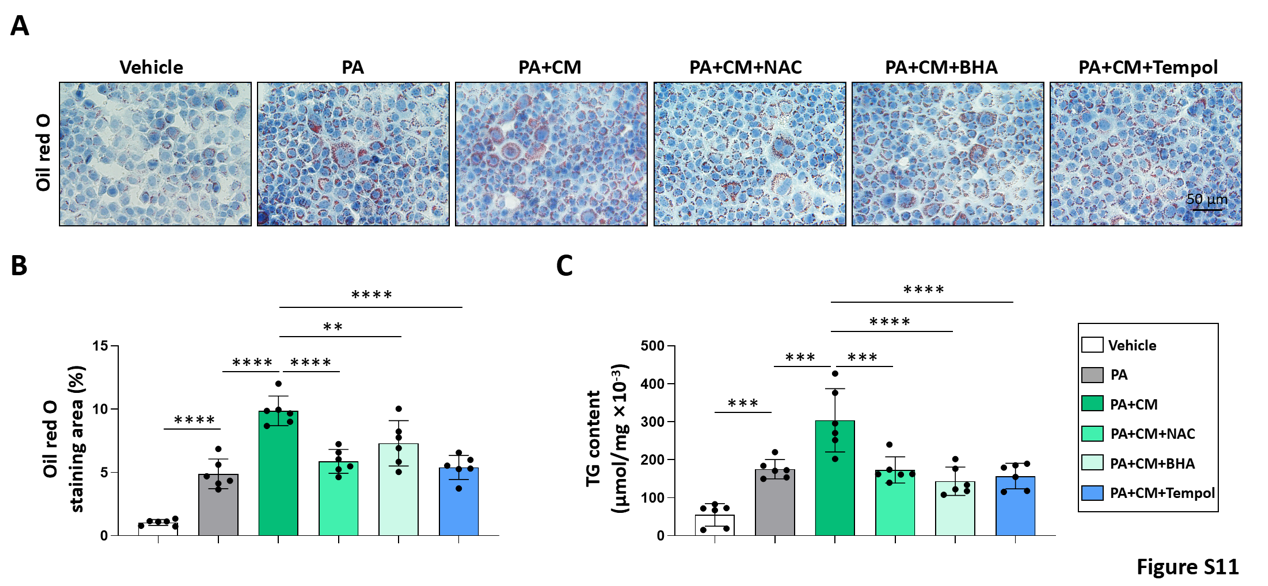


**Figure S11**

**N-acetylcysteine, Butylhydroxyanisole or Tempol attenuated lipid accumulation in hepatocytes *in vitro*.**

1. RAW264.7 macrophages were stimulated with LPS for 1 day, and the conditioned medium (CM) were harvested. Then AML12 hepatocytes were cultured with CM, in the presence or absence of ROS scavenger N-acetylcysteine (NAC, 2 mM), Butylhydroxyanisole (BHA, 100 µM) or SOD-mimetic agent Tempol (500 µM) in palmitic acid medium (PA, 200 µM) for 2 days. Lipid accumulation in AML12 cells was assessed using Oil Red O staining.

(B) The positive areas of Oil Red O staining in (A) were quantitatively compared.

(C) Triglyceride (TG) content in AML12 hepatocytes was measured. Bars = means ± SD, * P < 0.05, ** P < 0.01, *** P < 0.001, **** P < 0.0001.

**
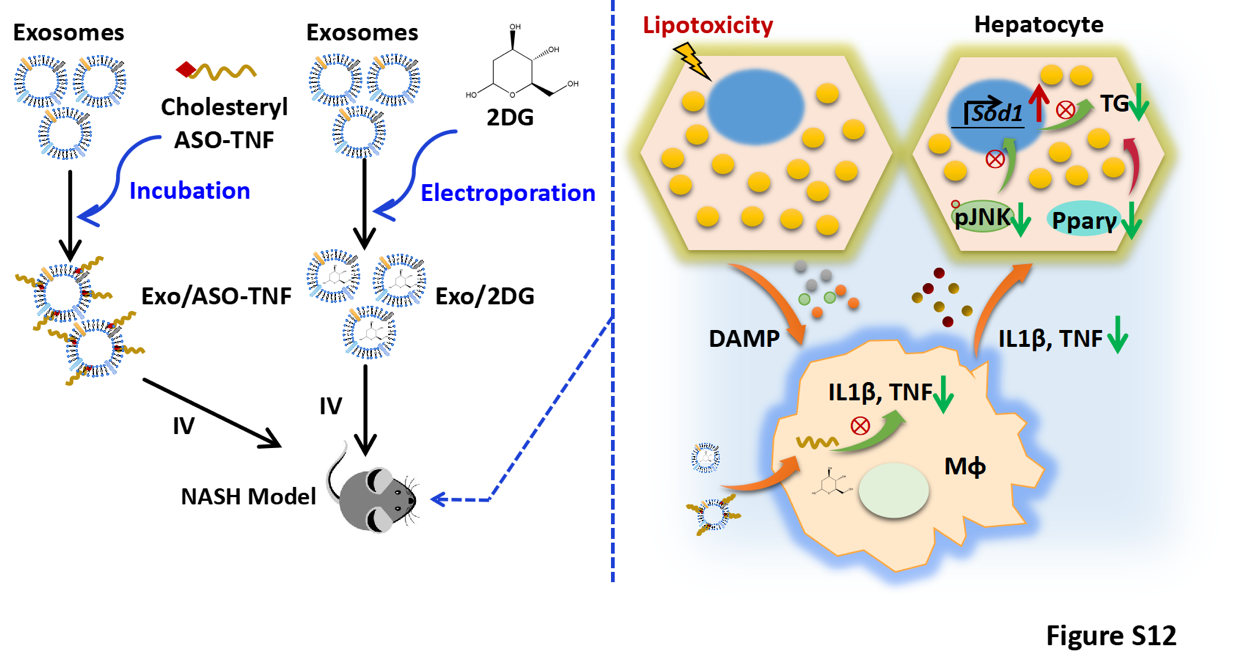
**

**Figure S12**

**Schematic summary of this study.**

During the progression of NAFLD, injured hepatocytes caused by over-accumulated lipid toxicity, released damage-associated molecular patterns (DAMPs), and then stimulated hepatic macrophages to release pro-inflammatory cytokines such as IL1β, TNF. And then IL1β and TNF in turn enhanced lipid accumulation in hepatocytes. On the other hand, exosomes injected intravenously were mainly distributed in the liver and taken up by hepatic macrophages. This indicates that exosomes represent a natural delivery system for the targeting of hepatic macrophages. In this study, we used exosomes targeting delivery of glycolysis inhibitor 2DG or antisense oligonucleotide against TNF and inhibited the expression of TNF and/or IL1β in macrophages. Finally, the infusion of Exo/ASO-TNF or Exo/2DG via tail vein ameliorated NASH in mice. Mechanistically, we found that infusion of Exo/ASO-TNF or Exo/2DG may play a therapeutic role by up-regulating the expression of Sod1.

**Table S1. Sequences of primers and other oligonucleotides used in the study**

| Name | Forward or sense (5’-3’) | Reverse or anti-sense (5’-3’) |
| --- | --- | --- |
| ASO-TNF | Cholesterol-5’-A*A*C*C*C*A*T*C*G*G*C*T*G*G*C*A*C*C*A*C-3’(Texas Red) |  |
| ASO-CT | Cholesterol-5’-T*C*A*A*G*C*A*G*T*G*C*C*A*C*C*G*A*T*C*C-3’ |  |
| β-actin | GGCTGTATTCCCCTCCATCG | CCAGTTGGTAACAATGCCATG |
| IL1β | GAAATGCCACCTTTTGACAGTG | TGGATGCTCTCATCAGGACAG |
| TNFα | CCCTCACACTCAGATCATCTTCT | GCTACGACGTGGGCTACAG |
| Pparγ | ATTCTGGCCCACCAACTTCGG | TGGAAGCCTGATGCTTTATCCCCA |
| Acc1 | GATGAACCATCTCCGTTGGC | GATGAACCATCTCCGTTGGC |
| Scd1 | TCTTCCTTATCATTGCCAACACCA | GCGTTGAGCACCAGAGTGTATCG |
| Fabp1 | TGGTCCGCAATGAGTTCACCCT | CCAGCTTGACGACTGCCTTGACTT |
| Cd36 | GGAGCCATCTTTGAGCCTTCA | GAACCAAACTGAGGAATGGATCT |
| Slc27a2 | ATGATCGGCCTTCACGGATG | CCCGGTCATTTGGTTTCTGC |
| Slc27a5 | TGTAACGTCCCTGAGCAACC | TAAGCCCACATTGCCCTCTG |
| Pparɑ | TATTCGGCTGAAGCTGGTGTAC | CTGGCATTTGTTCCGGTTCT |
| Cpt1ɑ | AGGACCCTGAGGCATCTATT | ATGACCTCCTGGCATTCTCC |
| Sod1 | TGTCCATTGAAGATCGTGTGAT | TCATCTTGTTTCTCATGGACCA |
| Apoa2 | AGCACAGAATCGCAGCACT | AGCTCCTTCCAGGCTACAGA |
| Cox6a1 | GAGGAGGGTTCAGCTCGGATG | TGAGGGTAGGCAACGAACG |
| Ndufa6 | TCTGCTGGTCATTAAGGGAAAGA | CATGCACCTTCCCATCAGGT |
| Icam1 | CAGTGGGTCGAAGGTGGTTC | CAGCCGAGGACCATACAGCA |
| Irak1 | CAGCCTACTCTGTGGGTGG | TGTTGGGAGTTTTGCCTCTGG |
| iNOS | GCAGAGATTGGAGGCCTTGTG | GGGTTGTTGCTGAACTTCCAGTC |
| IL6 | TAGTCCTTCCTACCCCAATTTCC | TTGGTCCTTAGCCACTCCTTC |
| IL12 | GGAAGCACGGCAGCAGAATA | AACTTGAGGGAGAAGTAGGAATGG |
| Arg1 | AGACAGCAGAGGAGGTGAAGAG | CGAAGCAAGCCAAGGTTAAAGC |
| Cd163 | CTGGCGGGTGGTGAAAACA | CAGCCGTTACTGCACACTG |
| IL10 | GCTCTTACTGACTGGCATGAG | CGCAGCTCTAGGAGCATGTG |
| Catalase | AGCGACCAGATGAAGCAGTG | TCCGCTCTCTGTCAAAGTGTG |
| miR-188-5p  mimics | 5’-CAUCCCUUGCAUGGUGGAGGG-3’-Cholesterol | CUCCACCAUGCAAGGGAUGUU |
| miRNA mimics NC | 5’-UCACAACCUCCUAGAAAGAGUAGA-3’-Cholesterol | UCUACUCUUUCUAGGAGGUUGUGA |
| miR-188-5p | 5’-CATCCCTTGCATGGTGGAGGG-3’ |  |
| U6 | 5’-GGATGACACGCAAATTCGTGAAGC-3’ |  |
| siSod1-Si#1 | GCAAUGUGACUGCUGGAAA(dT)(dT) | UUUCCAGCAGUCACAUUGC(dT)(dT) |
| siSod1-Si#2 | GGUCCAUGAGAAACAAGAU(dT)(dT) | AUCUUGUUUCUCAUGGACC(dT)(dT) |
| siSod1-Si#3 | GUUGUGUUGUCAGGACAAA(dT)(dT) | UUUGUCCUGACAACACAAC(dT)(dT) |
| siRNA-NC | UUCUCCGAACGUGUCACGU(dT)(dT) | ACGUGACACGUUCGGAGAA(dT)(dT) |

*, phosphorothioate

**Table S2. Antibodies used in this study**

| Antibody | Supplier | Cat.No | Purpose |
| --- | --- | --- | --- |
| IL1β | Proteintech | 26048-1-AP | WB |
| TNFα | Proteintech | 17590-1-AP | WB |
| Gapdh | Proteintech | 10494-1-AP | WB |
| ɑTubulin | Proteintech | 10068-1-AP | WB |
| Tsg101 | Proteintech | 28283-1-AP | WB |
| Alix | Cell Signaling Technology | 92880 | WB |
| Flotillin-1 | Cell Signaling Technology | 18634 | WB |
| Vdac-1 | Cell Signaling Technology | 4661 | WB |
| Pparγ | Proteintech | 16643-1-AP | WB |
| Sod1 | Proteintech | 10269-1-AP | WB |
| Catalase | Proteintech | 21260-1-AP | WB |
| SAPK/JNK | Cell Signaling Technology | 9252 | WB |
| pSAPK/JNK | Cell Signaling Technology | 4668 | WB |
| α-SMA | Servicebio | GB111364 | IHC |
| Col1α1 | Servicebio | GB11022-3 | IHC |
| F4/80 | Servicebio | GB113373 | IHC |
| Goat anti-Rabbit IgG (H+L)-HRP | Zhuangzhi Bio | EK020 | WB |
| Goat anti-Mouse IgG (H+L)-HRP | Zhuangzhi Bio | EK010 | WB |
| Goat anti-Rabbit IgG-HRP | Servicebio | GB23303 | IHC |
| Catalase | Proteintech | 21260-1-AP | WB |
| Mouse reactive inflammasome antibody sampler kit | Cell Signaling Technology | 20836 | WB |
| Goat anti Rabbit FITC | Jackson Immuno Research | 111-095-003 | IF |
